# Genetic heterogeneity in primary and relapsed mantle cell lymphomas: Impact of recurrent *CARD11* mutations

**DOI:** 10.18632/oncotarget.9500

**Published:** 2016-05-20

**Authors:** Chenglin Wu, Noel FCC de Miranda, Longyun Chen, Agata M. Wasik, Larry Mansouri, Wojciech Jurczak, Krystyna Galazka, Monika Dlugosz-Danecka, Maciej Machaczka, Huilai Zhang, Roujun Peng, Ryan D. Morin, Richard Rosenquist, Birgitta Sander, Qiang Pan-Hammarström

**Affiliations:** ^1^ Division of Clinical Immunology and Transfusion Medicine, Karolinska Institutet at Karolinska University Hospital, Huddinge, Sweden; ^2^ Beijing Genomics Institute, Shenzhen, China; ^3^ Division of Pathology, Department of Laboratory Medicine, Karolinska Institutet at Karolinska University Hospital, Huddinge, Sweden; ^4^ Department of Immunology, Genetics and Pathology, Science for Life Laboratory, Uppsala University, Uppsala, Sweden; ^5^ Department of Hematology, Jagiellonian University Collegium Medicum, Kraków, Poland; ^6^ Department of Pathology, Jagiellonian University Collegium Medicum, Kraków, Poland; ^7^ Faculty of Health Sciences, Jagiellonian University Collegium Medicum, Michalowskiego, Poland; ^8^ Department of Lymphoma, Tianjin Medical University Cancer Hospital and Institute, Tianjin, China; ^9^ Department of Medical Oncology, Sun Yat-Sen University Cancer Center, State Key Laboratory of Oncology in South China, Collaborative Innovation Center of Cancer Medicine, Guangzhou, China; ^10^ Department of Molecular Biology and Biochemistry, Simon Fraser University, Burnaby, Canada

**Keywords:** whole-exome sequencing, mantle cell lymphoma, relapse, CARD11, NF-κB inhibitor

## Abstract

The genetic mechanisms underlying disease progression, relapse and therapy resistance in mantle cell lymphoma (MCL) remain largely unknown. Whole-exome sequencing was performed in 27 MCL samples from 13 patients, representing the largest analyzed series of consecutive biopsies obtained at diagnosis and/or relapse for this type of lymphoma. Eighteen genes were found to be recurrently mutated in these samples, including known (*ATM, MEF2B* and *MLL2*) and novel mutation targets (*S1PR1* and *CARD11*). CARD11, a scaffold protein required for B-cell receptor (BCR)-induced NF-κB activation, was subsequently screened in an additional 173 MCL samples and mutations were observed in 5.5% of cases. Based on *in vitro* cell line-based experiments, overexpression of *CARD11* mutants were demonstrated to confer resistance to the BCR-inhibitor ibrutinib and NF-κB-inhibitor lenalidomide. Genetic alterations acquired in the relapse samples were found to be largely non-recurrent, in line with the branched evolutionary pattern of clonal evolution observed in most cases. In summary, this study highlights the genetic heterogeneity in MCL, in particular at relapse, and provides for the first time genetic evidence of BCR/NF-κB activation in a subset of MCL.

## INTRODUCTION

Mantle cell lymphoma (MCL) is a mature B-cell neoplasm that constitutes 5–7% of all lymphomas. MCL was considered to arise from naive B-cells that populate the mantle zone of lymphoid follicles, but recent findings advocate that antigen-experienced B-cells can also give rise to MCL [1–4]. Two main morphological subtypes of MCL, classical and blastoid, have been described and the latter is associated with a particularly poor prognosis for patients [5]. Clinically, most of the MCL cases show an aggressive disease course, with a limited duration of response to conventional chemotherapy and frequent relapses, resulting in an overall short survival time as compared to other B-cell lymphomas [6].

The initial oncogenic event in MCL, in the vast majority of cases, is a t(11;14)(q13;q32) translocation that juxtaposes the *CCND1* gene, encoding cyclin D1, to the immunoglobulin heavy chain (*IGH*) locus [7]. Mutations and/or deletions in the gene encoding ATM, a key regulator of the DNA damage-response machinery, have been identified in up to 50% of MCL cases and constitute one of the major secondary genetic events in MCL development [8]. Genetic alterations affecting other DNA damage response factors, including CHEK2 and P53, have also been described [9, 10]. Furthermore, *TNFAIP3* (*A20*), encoding a negative regulator of the NF-κB pathway, is frequently deleted in MCL [11].

Recently, a number of next-generation sequencing-based approaches have led to the discovery of novel oncogenic mechanisms in several types of mature B-cell lymphomas, including diffuse large B-cell lymphoma (DLBCL) [12–17], follicular lymphoma [18] and Burkitt lymphoma [19]. In MCL, RNA-sequencing revealed frequent mutations in *NOTCH1*, which were found to be associated with poor clinical outcomes [20]. Targeted re- sequencing of 18 genes further showed that *UBR5*, an E3 ubiquitin ligase-encoding gene, was mutated in 18% of the MCL samples analyzed [21]. Recently, two whole-genome and/or whole-exome sequencing (WES) studies on 29 and 56 MCL cases, respectively, identified additional mutated genes, including those encoding chromatin modifiers (*WHSC1*, *MLL2*, *MLL3*, *MEF2B* and *SMARCA4*), a cell adhesion factor (*FAT4*), cell cycle regulators (*SMC1A* and *POT1*), an anti-apoptotic factor (*BIRC3*) and *NOTCH2* [22, 23].

As the clinical course of MCL patients is variable, and the genetic landscape of tumors might be quite heterogeneous, sequencing of additional tumor genomes can provide further insight on deregulated pathways that are involved in this disease. Furthermore, the genetic mechanisms linked to relapse and therapy resistance, are still largely unknown. To this end, we compared the mutation landscape of a set of primary (at diagnosis) and relapse MCL tumors samples derived from the same patients.

## RESULTS

### The landscape of mutations in the coding genome of primary and relapse MCL

**Figure 1:**
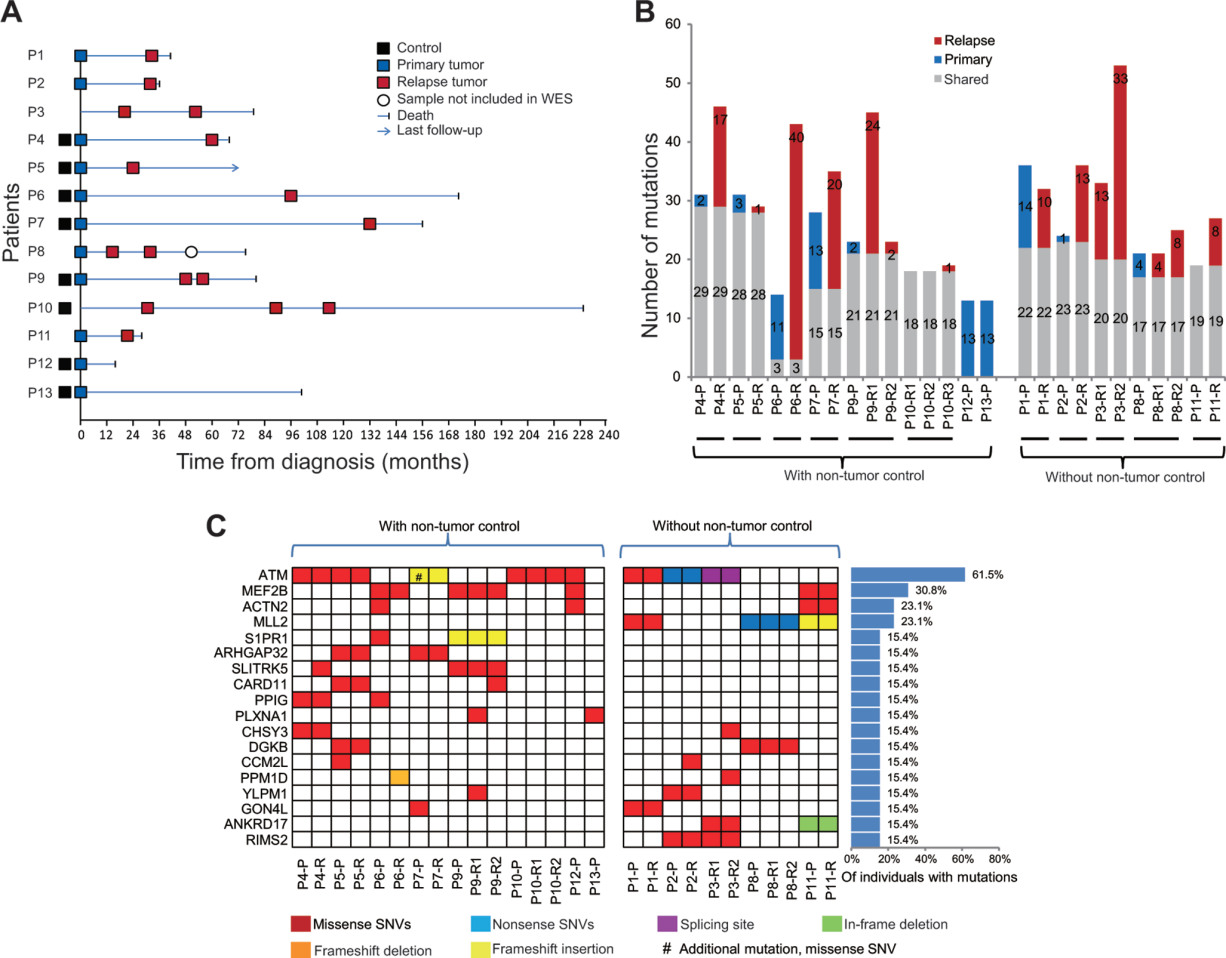
Clinical timelines and landscape of somatic mutations in mantle cell lymphoma (MCL) patients (**A**) Sample information with disease event timelines for the thirteen MCL cases included in the WES study. (**B**) Number of somatic, non-silent variants identified in each MCL tumor sample (blue, only detected in the primary tumor; red, only detected in the relapse tumor; gray, shared by paired primary and relapse MCL). P, primary tumors; R, relapse tumors. (**C**) Recurrently mutated genes in 13 patients with MCL. Genes are listed only when they were affected by non-silent SNVs or indels in at least two MCL patients. #, additional missense SNVs were observed in the marked sample.

WES was performed on 27 tumors (11 primary and 16 relapse) and 8 matched non-tumor- samples derived from 13 MCL patients (Figure 1A and Table S1). Together, a mean sequence depth greater than 76x was achieved and on average, 88.0% (range, 81.1–94.3%) of bases constituting the human coding genome were covered by at least 10 sequencing reads (Table S2).

An overview of data analysis strategy is shown in Figure S1. Somatic (tumor-specific) mutation analysis was first performed in 16 tumors (7 primary and 9 relapse) for which paired non-tumor control samples were available (Figure S1, green shadow). Altogether, 274 somatic, non-synonymous variants were identified in 254 genes. From these, 56 and 105 variants were specific for primary or relapse samples, respectively, whereas 113 variants were shared between primary and relapse samples (Figure 1B, left panel, “with non-tumor control”; Table S3).

Variants presented only in the primary or relapse samples were subsequently determined in 11 tumors (4 primary and 7 relapse) for which the respective non-tumor control samples were not available (Figure S1, blue shadow). Nineteen non-synonymous SNVs were specifically identified in the primary samples and 83 non- synonymous SNVs as well as 6 indels were only observed in relapse cases (Table S3). These alterations were considered as of likely somatic origin since germline variants are expected to be present in all tumor samples, except in case of loss of genetic material. Additional somatic variants in these tumors, including 94 SNVs and 7 indels, were identified by filtering the mutations that are shared by the paired primary and relapse samples (Table S4; Figure S1, pink shadow). To exclude mutations with potential germline origin, only variants associated with previously reported, recurrently mutated genes in MCL and DLBCL [12–14, 20–24] and with minor allele frequency < 0.1% in 1000 genome were considered. Altogether, 209 of potentially somatic variants were observed in 194 genes in the 11 tumors samples without paired non-tumor controls (Figure 1B, right panel).

### Recurrently mutated genes in MCLs

By joining all analysis for tumor-related variants, 454 non-silent SNVs and 29 indels, somatic or putatively somatic, were identified in the 27 MCL samples. Twenty-five recurrent mutation targets, i.e. genes affected by non-silent SNVs and indels in at least two patients, were identified (Table S5). Seven genes (*TTN*, *FAT1*, *PKHD1*, *PCLO*, *FLG*, *ABBA13* and *DNAH5*) that are highly mutated/damaged in germline genome from healthy individuals at a population level were subsequently excluded [25], and the remaining 18 genes are nominated as recurrently mutated genes for this patient cohort (Figure 1C). Except for *ATM*, *MEF2B* and *MLL2*, these genes may represent novel mutation targets in MCL. *S1PR1* was found mutated in four tumor samples derived from two patients. It encodes for the sphingosine-1-phosphate receptor 1, which has been shown expressed in mantle zone B cells and MCL [26, 27]. Mutations in the *CARD11* gene, which is known to be recurrently targeted in DLBCL and in splenic marginal zone lymphomas [28, 29], were discovered in three tumor samples derived from two patients. A mutation in the *CARD11* gene has previously been described in 1/29 primary MCL cases [22], but to the best of our knowledge, recurrent mutations in the *CARD11* gene have not been reported in MCL cell line or MCL cases in the literature. Importantly, however, our re-analysis of exome data from the aforementioned studies uncovered a limited number of mutations affecting *S1PR1* in each cohort. In the first cohort [23], one patient harbored a frameshift deletion and in the second cohort [22], one patient harbored a missense mutation (R324L) and a second mutation harbored an in-frame insertion (G122_S123insN). Two patients with *CARD11* mutations were also observed in the second cohort (P100Q and K215T). Other recurrent mutation targets that have been implicated in different types of cancers included *PPM1D* and *GON4L* (Table S5).

### CARD11 mutations in MCLs

**Figure 2:**
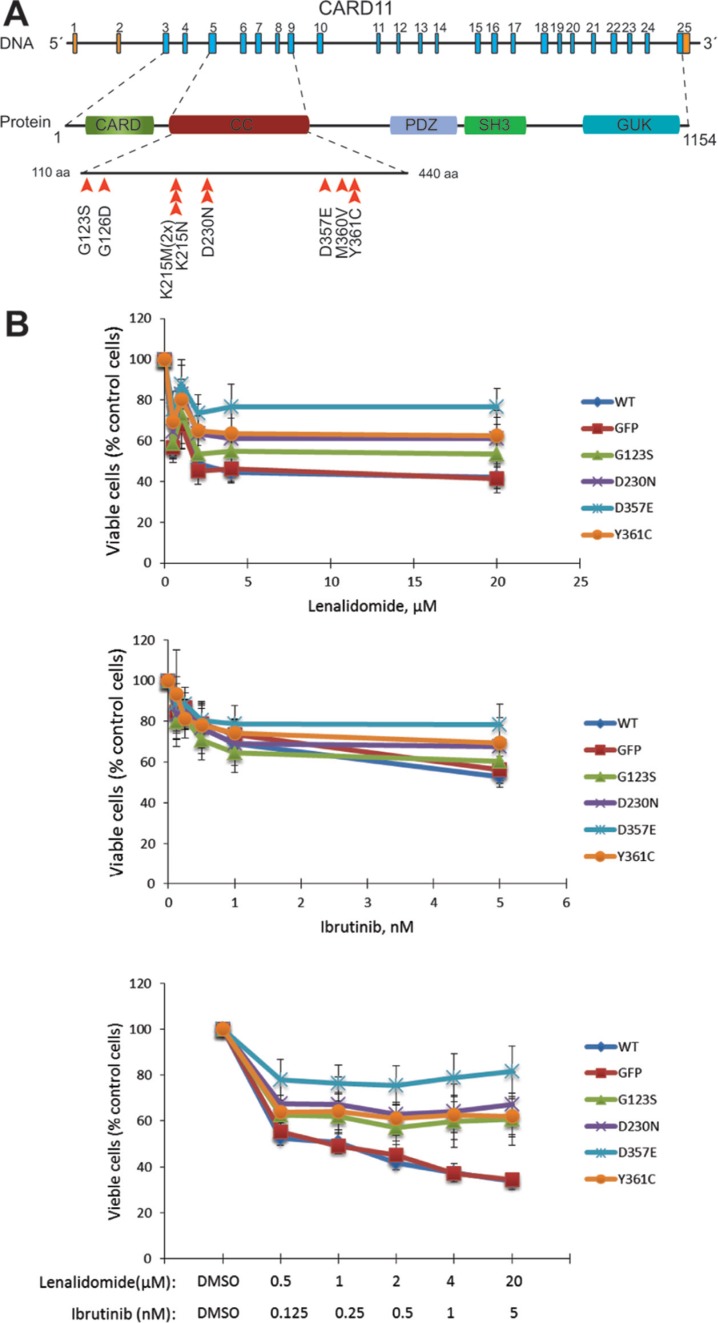
Variants identified in *CARD11* gene resulted in resistance to ibrutinib and lenalidomide treatments (**A**) The distribution of *CARD11* variants identified in the discovery and the expanded cohorts of MCL patients. Altogether, 200 Swedish MCL samples from 179 patients were screened by WES and/or Sanger sequencing. The screening effort was focused on the coiled-coil domain of *CARD11* (from exon 4 to exon 8). The positions of variants in coiled-coil domain of *CARD11* are indicated by arrowheads. One mutation (p.K215M) was found in two samples of P5. (**B**) Drug inhibition assay in MCL cell lines with over expression of wild-type or the mutant forms of *CARD11* with adenovirus system. The MCL cell line Rec-1 was treated with lenalidomide, ibrutinib or with a combination of the two drugs for four days at the concentrations indicated. The average percentages of viable cells from four independent experiments were plotted and the error bars indicate the standard errors. The difference is significant for mutant D357E vs. wild-type (*p* = 0.04921), when treated with ibrutinib (5 nM); mutant D357E vs. wild-type or GFP control (*p* = 0.02058 and 0.02057), when treated with lenalidomide (20 μM); mutant D357E vs. wild-type (*p* = 0.02419) when treated with ibrutinib (5 nM) and lenalidomide (20 μM) (paired-sample sign test).

Due to the known importance of activating mutations in the coiled-coil (CC) domain of the CARD11 protein in the pathobiology of DLBCL, the observation of mutations in our analysis of two published cohorts [28, 30] and in a third unpublished exome cohort of unrelated Canadian MCL samples, this gene domain was further screened by Sanger sequencing in an extended cohort of MCL. Altogether, 5.5% (11/200) of MCL samples or 5.6% (10/179) of the MCL patients were affected by mutations in the CC domain of CARD11 (Figure 2A and Table S6), including the mutations identified by WES (in P5 and P9, Table S6). The mutation frequency in *CARD11* in our MCL cohort is thus about half as that observed in previous described DLBCL cohorts [13, 28]. Notably, some of the mutations identified in MCL samples are identical to those observed in DLBCL, including G123S, K215M and D230N. The mutations status in our cohort was not associated with any of the morphological subtypes and did not affect the overall survival of patients (Table S7). But notably, *CARD11* mutations tended to be more frequent in relapse tumors, although not to a significant degree (36.4% (4/11) at relapse vs. 22.9% (43/188) in primary MCL, *p* = 0.2190, Table S7).

Activating mutations in the CC domain of *CARD11* may result in a constitutive activation of B-cell receptor (BCR)/NF-κB signaling and render the mutant cells resistant or sensitive to some of the BCR/NF-κB inhibitors (targeting molecules upstream or downstream of CARD11, respectively) [28]. Six MCL cell lines (Granta519, JVM2, Rec-1, Mino, Jeko-1 and Z-138) were characterized by WES to identify *CARD11* mutations or related mutations and were also tested *in vitro* by treatment with ibrutinib (a BTK inhibitor) and/or lenalidomide (a NF-κB inhibitor). The proliferation of four MCL cell lines (Rec-1, Mino, Z-138 and Jeko-1) was inhibited to various degrees by either ibrutinib or lenalidomide or by combination of two drugs (Figure 2 and Figure S2). Although no *CARD11* mutations were found in any of the 6 MCL lines sequenced, other mutations affecting the BCR or NF-κB pathway might explain the observed treatment responses (Table S8). For instance, i) a damaging mutation in *NFKBIB* was identified in the cell line Rec-1, which was sensitive to both drugs, especially lenalidomide (Table S8), and ii) a nonsense mutation in the gene encoding TRAF2, a factor involved in the non-canonical NF-κB pathway, was found in Z-138, which might result in ibrutinib resistance [31], but at the same time did not affect sensitivity to lenalidomide (Table S8 and Figure S2).

Wild-type and four *CARD11* mutants (G123S, D230N, D357E and Y361C) were subsequently overexpressed in the ibrutinib/lenalidomide-sensitive cell line Rec-1 and insensitive cell lines Granta519 and JVM2. The expression level of wild-type CARD11 and its different mutants was monitored by western blot analysis (Figure S3), and the transfection efficiency was estimated by expression of GFP. *CARD11* mutants, but not the wild-type protein, desensitize the Rec-1 cell line to ibrutinib and lenalidomide treatment, and the difference is significant especially when the D357E mutant was introduced and the combination of ibrutinib/lenalidomide with higher concentrations were introduced (Figure 2B). The other two MCL cell lines (JVM2 and Granta519) remained insensitive to both drugs (Figure S4). These two cell lines are positive for Epstein-Barr virus (EBV), an activator of the alternative NF-κB pathway, and therefore, may render the cells resistant to BCR/NF-κB pathway inhibitors. Taken together, acquiring *CARD11* mutations might result in resistance to inhibitors targeting the BCR/NF-κB signaling pathway.

### Genetic alterations associated with tumor relapse are heterogeneous

Altogether, 194 somatic alterations were specifically identified in the relapse samples, including 183 non-synonymous SNVs and 11 indels (Figure S1 and Table S3B). Thus, a median of 12 variants (range, 0–40) per case has been acquired in the relapse MCL samples. These mutations however, are largely non-recurrent, with only one gene,*PPM1D*, involved in p53 signaling, mutated in two relapse samples (P3-R1 and P6-R). Affected genes were subsequently assigned to pathways with WebGestalt analysis [32] and KEGG [33], and the MAPK, p53 and ErbB signaling pathways were shown to be enriched for genes affected by mutations in the relapse samples (Figure S5 and Table S9). By performing literature mining, we furthermore identified that DNA repair, NF-κB and Wnt signaling pathways might also be associated with genetic lesions present at relapse (Figure S5).

### Clonal evolution in different MCL paired tumors

In order to determine whether tumors collected at different time points from the same patient were clonally related, the coding junctions of *IGHV-IGHD-IGHJ* rearrangements in altogether 25 tumor samples from 11 patients were amplified. V gene sub-families including *IGHV3* and *IGHV4* were preferentially used as previously reported [34]. The dominant tumor cell clones, based on the V(D)J coding joints, were identical in paired samples from the same individual in all patients, suggesting that common precursor for the primary and relapsed tumors (Table S10). The *IGHV* genes were unmutated/minimally mutated in most of the samples, except in a few cases (P1- P, P5-P and P5-R, Table S10).

**Figure 3:**
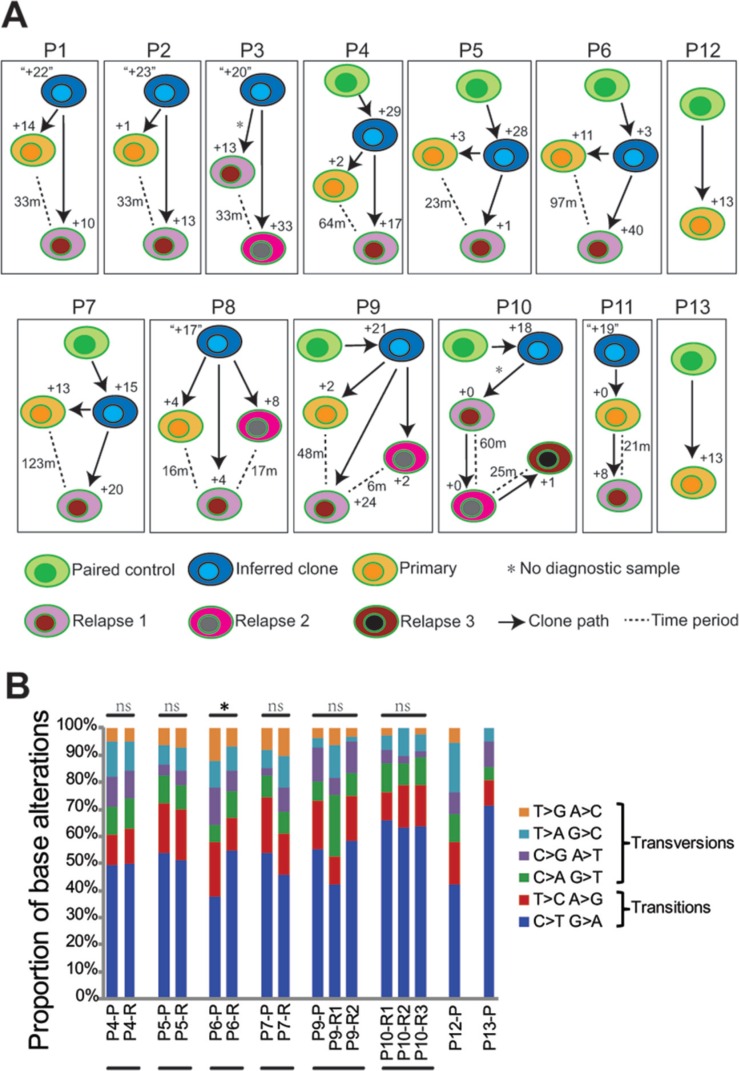
Clonal evolution of MCL based on mutational analyses (**A**) Clonal evolution of relapse samples analyzed based on analysis of non-silent mutations. Arrows indicate the clonal pathway between samples. In each case, the analyzed primary MCL tumor is represented by an orange cell, the relapse tumor by a pink cell, and second relapse tumor by a dark-pink cell (available in P3 and P8-P10), and third relapse tumor by a brown cell (P10). The non-tumor control samples are represented as green cells and inferred clones are shown as blue cells. The number of genetic lesions acquired in the progression of MCL is depicted. The numbers with double quotation marks indicate that these shared mutations were achieved by filtering with mutations described in previous literature on DLBCL and MCL (see Materials and Methods). The time interval between primary and relapse or between the two relapses was indicated as number of months (m) beside the dotted line. Asterisks (for P3 and P10) indicate that the primary sample is not included in this study. (**B**) The pattern of somatic mutations identified in each primary and relapsed MCL tumors with paired non-tumor controls. Samples derived from the same patients were subgrouped. Mutation types that significantly differ in a given relapse sample as compared to the corresponding primary sample (or the first relapse sample if primary sample is not available) is labeled with asterisk. Wilcoxon signed-rank test was used for statistical analysis. ns, not significant; *p < 0.05.

Tumor clonal evolution may occur through a branched or a linear model [35]. Based on the number of non-silent SNVs and indels that were specific or shared between the paired tumor samples, the history of clonal evolution in each sample was further inferred. In most of cases (P1-P9), unique genetic lesions were observed in both the primary and relapse MCL samples, and this scenario is consistent with a predominantly branched evolutionary model (P1-P9 in Figure 3A). For example, a mutation in *ATM* (p.G2063E) was observed in P7-P but not in P7-R (Figure 1C), conversely *PLCG1* was mutated only in P7-R. Nevertheless, in two cases, all lesions identified in the primary samples were maintained in the relapse tumors (P10 and P11), which is consistent with a linear pattern of evolution (P10-P11 in Figure 3A). The copy number changes estimated by the WES data in general support the above findings.

The composition of base substitutions was further analyzed for primary and relapsed samples with matched non-tumor controls. For this analysis, all somatic mutations including silent mutations as well as mutations in the non-coding regions were included. Mutation patterns were altered in the relapse samples as compared to the corresponding primary samples in three patients (P6, P7 and P9), but only to a statistically significant level in P6 (*p* = 0.0355, Wilcoxon signed-rank test, Figure 3B). Interestingly, all three patients had been treated with autologous stem cell transplantation (ASCT) (Table S1). As the primary and relapse tumors in these three patients shared the same V(D)J sequences, like the other patients, this may suggest that although the relapse tumor is likely derived from the same precursors/stem cells as the primary tumor, the transplantation procedure might lead to selection of a different sub-clone of precursor cells or influence the pattern of mutations acquired at the later stage.

## DISCUSSION

MCL is treated with front-line combination chemotherapy or intensive chemo-immunotherapy followed by stem-cell transplantation as consolidation [6]. Most of the patients initially respond to these therapies, but tumor relapse invariably occurs and the disease remains incurable. In this study, WES was performed in paired primary and relapsed MCL tumors and genetic changes associated with therapy resistance and disease relapse were identified.

The genomic alterations at diagnosis and progression have so far only been studied in two MCL cases by NGS, showing linear or branched pattern of clonal evolution, respectively [22]. In our study, with 11 cases studied, a branched evolutionary pattern was most prevalent. Furthermore, we discovered that MCL relapse tumors acquire on average 12 genetic lesions. However, these lesions are very heterogeneous, and most often non-recurrent, suggesting that multiple/different mechanisms might be involved in disease progression and this may contribute to the difficulties in obtaining long-lasting remissions in MCL patients. Moreover, specific therapy might also affect the clonal evolution patterns observed at relapse. For instance patients treated with ASCT had the most diverged primary/relapse clones. Our study, although by far the largest NGS analysis on paired primary/relapse samples, suggests that a substantially higher number of cases will be required in order to identify potential recurrent genetic events that are associated with disease relapse.

CARD11, a scaffold protein that forms a complex with BCL10 and MALT1 [36], is required for antigen receptor-induced NF-κB activation in B- and T-cells [28]. The expression of *MALT1* but not *BCL10* increased when several mutations in CARD11 were expressed in MCL cell lines (data not shown). Gain-of-function mutations in *CARD11* have been associated with lymphomas including DLBCLs and marginal zone lymphomas [28, 30]. In this study, recurrent mutations were observed in the coiled-coil domain of CARD11 in MCL, providing genetic evidence for a pathogenic role of the chronic activation of BCR/NF-κB signaling in these tumors. Ibrutinib, a BTK inhibitor, has shown encouraging anti-tumor activity against various B-cell malignancies including MCL [37]. However, as CARD11 is located downstream of BTK in the signaling cascade, ibrutinib may not have an effect on MCL cases that carry *CARD11* mutations. Lenalidomide, a drug that alone, or in combination with ibrutinib, can inhibit the growth of ABC-DLBCL cell lines by downregulating transcription factors IRF4 and SPIB, which in turn amplify the NF-κB activity [38], also showed an effect on a several of the MCL cell lines investigated. The function of lenalidomide seems to require the expression of the wild-type form of CARD11 [38]. In support of this, by introducing the mutant forms of CARD11, the sensitive cell line Rec-1 became resistant to treatment by lenalidomide or the combination of lenalidomide and ibrutinib. A recent study also showed that intact CARD11 is required for the activity of the pan-protein kinase C (PKC) inhibitor sotrastaurin in MCL lines [31]. Thus, mutations in *CARD11*, which occurred in almost 6% of the patients studied, would result in insensitivity to several of the inhibitors that are currently tested in the clinic. Additionally, genetic lesions in the components of the alternative NF-κB signaling pathway, including mutations in *TRAF2*, *BIRC3* and *MAP3K14* (NIK), were recently shown to be associated with resistance to ibrutinib [31]. Notably, a point mutation in *MAP3K14* was identified in a MCL sample (P13-P), which further suggests that genetic characterization of the tumor genome will be important for the choice of therapeutic strategy for MCL patients.

In summary, we have demonstrated that at the coding genome level, MCL relapse is associated with acquisition of new genetic lesions and in most of cases, the primary and relapse tumors seem to arise from a common progenitor, following a branched clonal evolutionary model. We have also identified novel mutational targets in MCL, exemplified by *CARD11*, which has important functional properties that provide new insights of the pathogenesis of MCL and may offer new therapeutic strategies.

## MATERIALS AND METHODS

### Patient samples

Thirteen Swedish MCL patients were included in the current WES study. Twenty-seven tumors (11 primary and 16 relapse cases) and 8 paired-normal tissue samples derived from these patients were analyzed (Figure 1A). Clinical and pathological information on all samples is presented in Table S1. Mutation prevalence in *CARD11* was determined in altogether 200 MCL samples from 179 Swedish patients diagnosed between 1998 and 2013, for which information on age, sex, stage, cytological subtype and survival status were available. Genomic DNA was extracted from peripheral blood, frozen tumor samples, or viability frozen cells using DNeasy Blood & Tissue Kit (Qiagen, Hilden, Germany) or GenElute Mammalian Genomic DNA Miniprep Kit (Sigma, Taufkirchen, Germany), following the manufacturers’ instructions. The institutional review boards at Karolinska Institutet and Uppsala University approved the study.

### Exome sequencing and analysis pipeline

The coding genomes of tumors and respective non-tumor control samples were captured by Agilent's SureSelect Human All Exon Kit and libraries were sequenced by Illumina's HiSeq 2000 system as described previously [15]. The generated sequences were aligned to the reference genome (hg19) using Burrows-Wheeler Aligner (BWA) [39].

Single nucleotide variants (SNVs) and insertions/deletions (indels) were identified by Varscan2 and GATK, respectively [40, 41]. For the paired tumor and non-tumor normal samples, the latter was served as a reference for the detection of somatic (tumor-specific) variants and a paired mode was used. Somatic variants were considered if represented in at least 10% of the sequencing reads in the tumor sample and in less than 2% of sequencing reads of the corresponding reference sample. For primary/relapse tumor pairs, without corresponding non-tumor normal samples, the variants specific for primary or relapse tumors were determined using their corresponding paired-tumor sample as a reference, following the above criteria for the identification of somatic mutations. For determination of shared variants derived from these primary/relapse tumor pairs (without non-tumor controls), all SNPs annotated in dbSNP135 and variants with population frequency greater than 0.1% in the 1000 Genomes (1000g2012feb) and Esp6500 Exomes datasets were subtracted. Furthermore, variants that were not associated with previously reported, recurrently mutated genes in MCL and DLBCL were excluded [12–14, 20–24]. This may underestimate the number of somatic mutations in these samples, however, will help to avoid including of rare germline mutations. Population-specific frequencies of SNVs discovered by next-generation sequencing were retrieved from 1000 Genomes and Esp6500 Exomes with the aid of VCFtools and Tabix [12, 42]. All SNVs and indels detected by bioinformatics analysis underwent visual inspection using IGV [43].

All mutated genes were included for pathway analysis with annotations performed using the KEGG [33] and WebGestalt tools [32]. Significance values were obtained from hypergeometric test (rawP) after multiple test adjustment [32].

After normalization of overall sequencing depths, DNA copy-number variations (CNVs) were called in tumors with reference to matched controls using ExomeCNV [44] and annotated by GISTIC2.0 to identify significant regions of amplification and deletion [45].

### Sanger sequencing

The coding regions of CC domain of *CARD11* were analyzed in altogether 179 MCL samples (including 6 samples that have been sequenced by WES) by Sanger sequencing. In addition, 29 mutations in other genes, identified by WES, were chosen for validation by Sanger sequencing and all alterations could be confirmed (Table S3B–S3C). PCR and sequencing primers were designed with NCBI/Primer-BLAST [46] and primer sequences are available upon request. The resulting PCR products were purified by using the Qiaquick PCR purification kit (Qiagen, Hilden, Germany) and sequenced at Macrogen Inc. (Seoul, Korea) or Eurofins MWG Operon (Ebersberg, Germany).

### Preparation of recombinant adenoviruses

AdEasy Adenoviral Vector System kit (Stratagene, Waldbronn, Germany) was used to prepare recombinant adenoviruses containing cDNA of wild-type or four *CARD11* mutants (G123S, D230N, D357E and Y361C) by homologous recombination [47]. Briefly, cDNA of wild-type or mutant *CARD11* was subcloned into pShuttle-CMV vector provided in the kit. The resulting plasmid was co-transformed with adenoviral backbone plasmid (pAdEasy-1) into *E. coli* BJ5183 cells. Recombinant adenoviruses were produced in HEK293 cells. The expression of GFP (AdGFP) was used as a marker for monitoring infection efficiency by calculating the percentage of GFP positive cells with a Zeiss Primovert microscope. The expression of different *CARD11* constructs was furthermore confirmed by western blot.

### Cell proliferation assay

Six MCL cell lines (Granta519, Jeko-1, Z-138, Rec- 1, Mino and JVM2) were included in the cell proliferation assay and were also analyzed by WES (Table S2). Cells were plated at a density of 8000 cells per well in 96-well plates. For overexpression of wild-type or mutant forms of *CARD11*, three MCL cell lines (Granta519, JVM2 and Rec-1) were infected with recombinant adenoviruses during cell plating and were incubated in a cell culture incubator at 37°C for 48 hours before the drug treatment. For each well, lenalidomide (0–4 μM, CC-5013, Selleckchem, Munich, Germany) and/or ibrutinib (0–1 nM, PCI-32765, Selleckchem) were added (final volume 200 ml). After 4 days of treatment, cell viability was evaluated by CellTiter 96^®^ AQueous one solution cell proliferation assay (Promega, Madison, USA) according to the manufacturer's instructions. Background absorbance was subtracted using a medium-only control.

### IGHV-IGHD-IGHJ rearrangements

To determine whether the primary and relapse samples derived from the same patient were clonally related, rearranged *IGHV*-*IGHD*-*IGHJ* genes were amplified from the tumor DNA samples as described previously [48]. *IGHV* gene usage, somatic mutations in the *IGVH* genes, length and composition of the CDR3 regions were analyzed by the IMGT/V-QUEST tools [49].

### Statistical analysis

Fisher's exact test or χ^2^ test was used for group comparisons. The Wilcoxon signed-rank test was used for the comparison of somatic mutation pattern between primary and relapse tumors from individual patient. Overall survival was measured from date of diagnosis to date of death or last follow-up. A *p* value of < 0.05 was considered statistically significant.

## SUPPLEMENTARY MATERIALS FIGURES AND TABLES


