# Supplementary material for: Genetic heterogeneity in primary and relapsed mantle cell lymphomas: Impact of recurrent CARD11 mutations
Source: Oncotarget. 2016 May 20;7(25):38180–90. doi: 10.18632/oncotarget.9500 (PMC5122381; doi:10.18632/oncotarget.9500)
Supplement: Supplementary file 1 [file oncotarget-07-38180-s001.pdf]

Genetic heterogeneity in primary and relapsed mantle cell lymphomas: Impact of recurrent *CARD11* mutations

Supplementary Materials

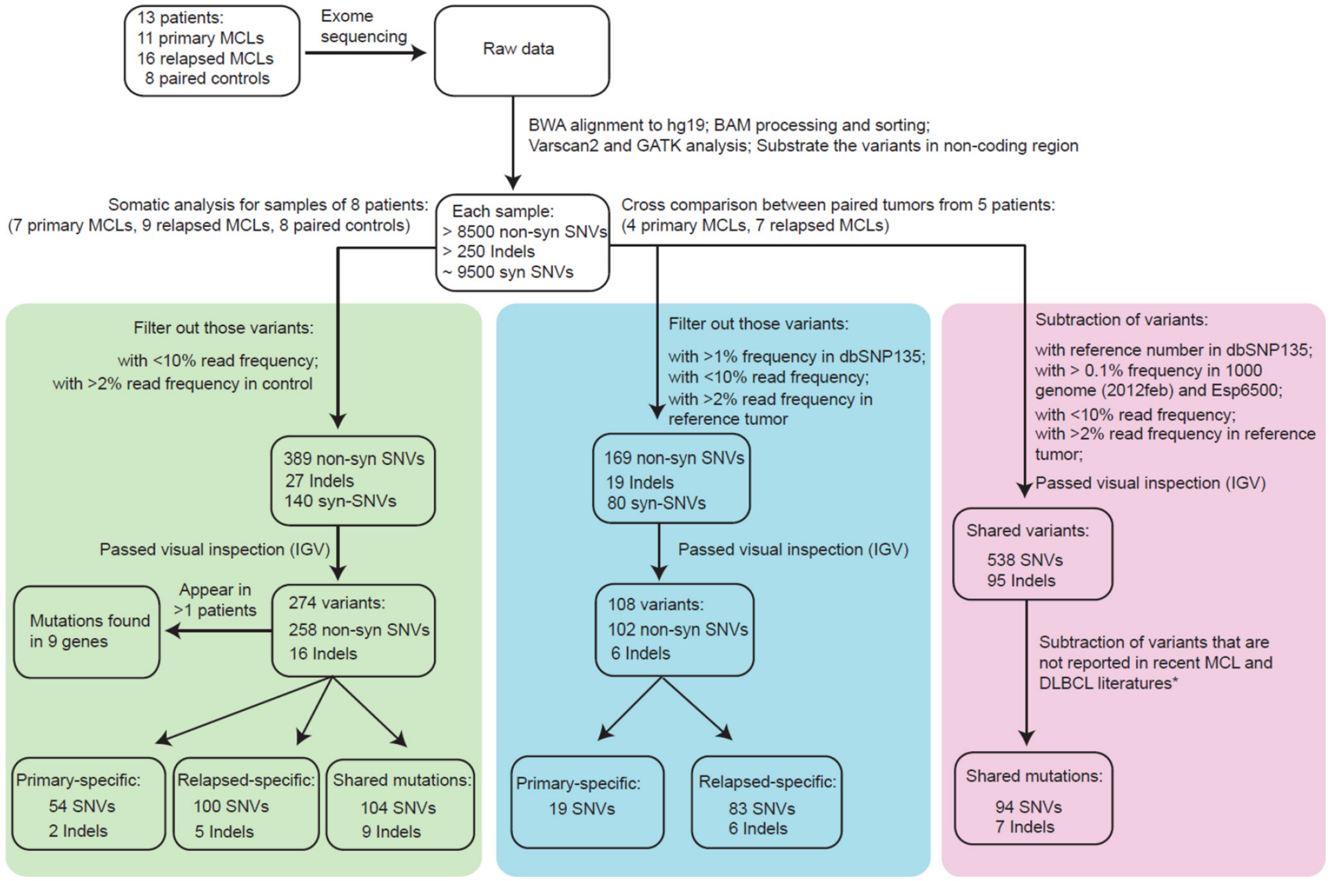

Supplementary Figure S1: Overview of data analysis strategy.

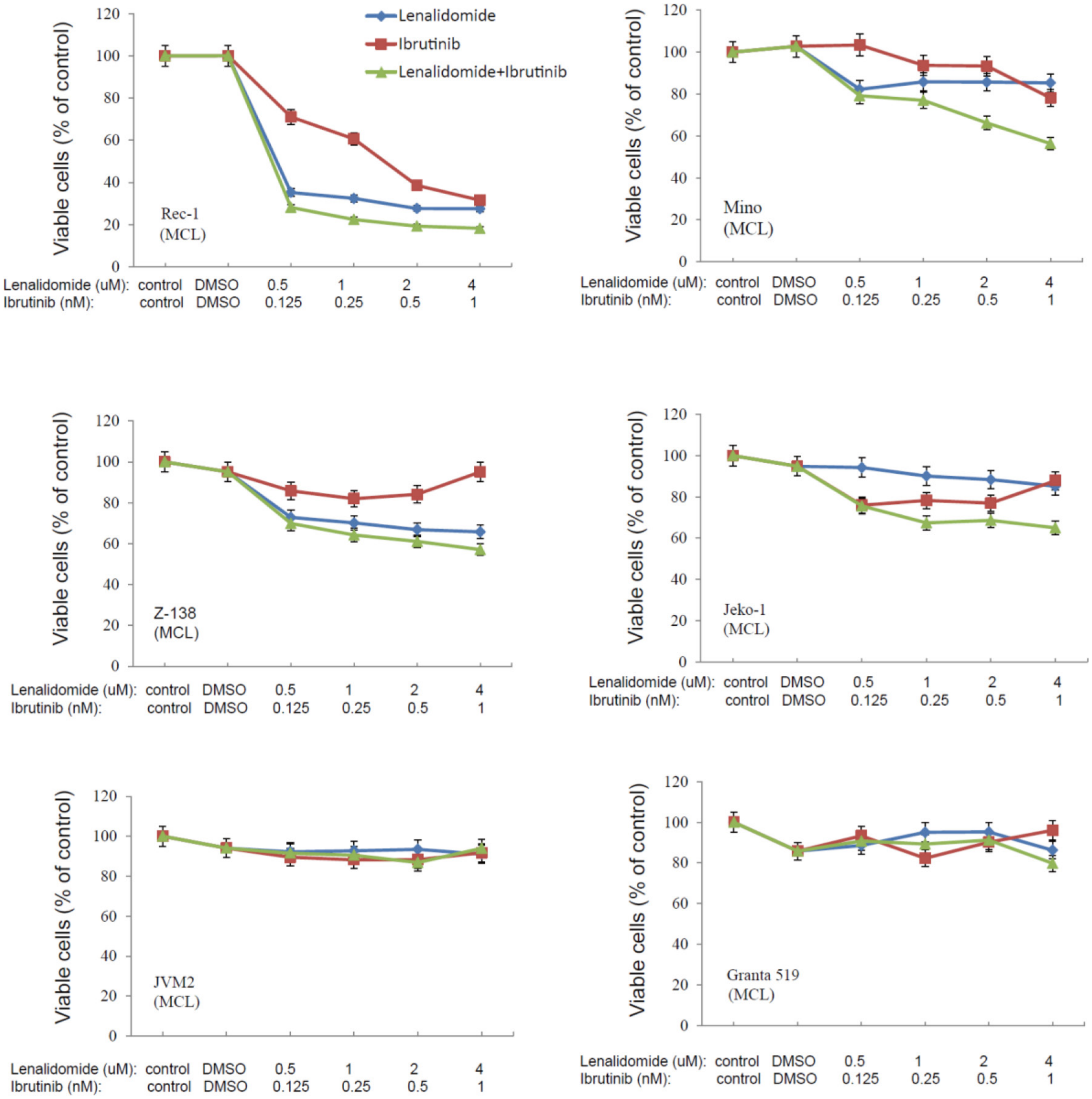

Supplementary Figure S2: Drug inhibiting assay in MCL. Six MCL cell lines (Rec-1, Mino, Z-138, Jeko-1, JVM2 and Granta519), were treated with lenalidomide, ibrutinib or with the combination of the two drugs for four days at the concentrations indicated. The concentrations of lenalidomide and ibrutinib are similar as previous described for ABC-DLBCL [1]. Jeko-1, Mino and Rec-1 are sensitive to both ibrutinib and lenalidomide to various degrees, Z-138 is sensitive to lenalidomide, whereas JVM2 and Granta519 are insensitive to both drugs. Control is the respective cell line without drug or DMSO treatment. Error bars represent the SEM of triplicates.

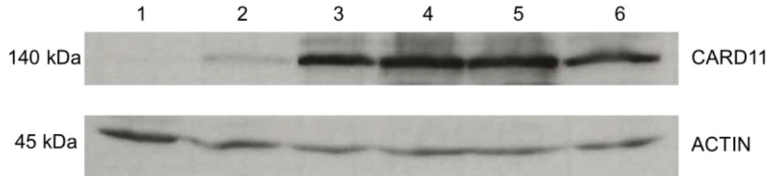

Supplementary Figure S3: Western blot for determining the expression of different *CARD11* constructs in HEK293 cell line. 1, Adenovirus without any insertion; 2, virus with *CARD11* G123S mutant; 3, virus with *CARD11* D230N mutant; 4, virus with *CARD11* D357E mutant; 5, virus with *CARD11* Y361C mutant; 6, virus with *CARD11* wild-type.

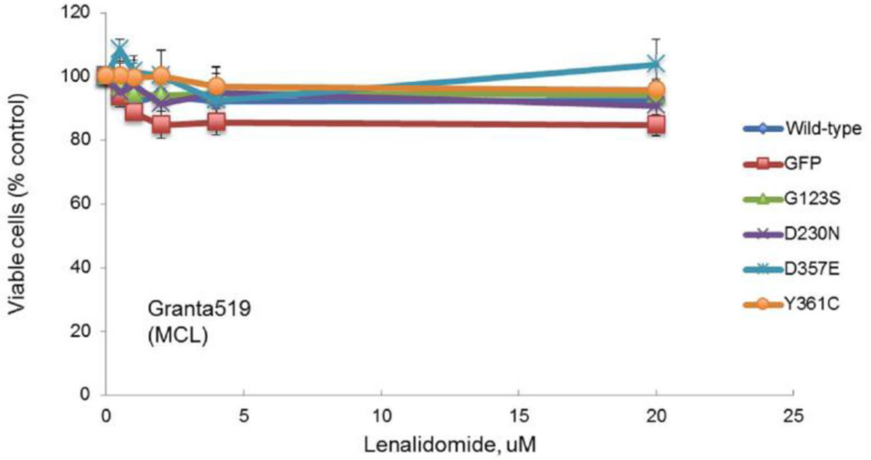

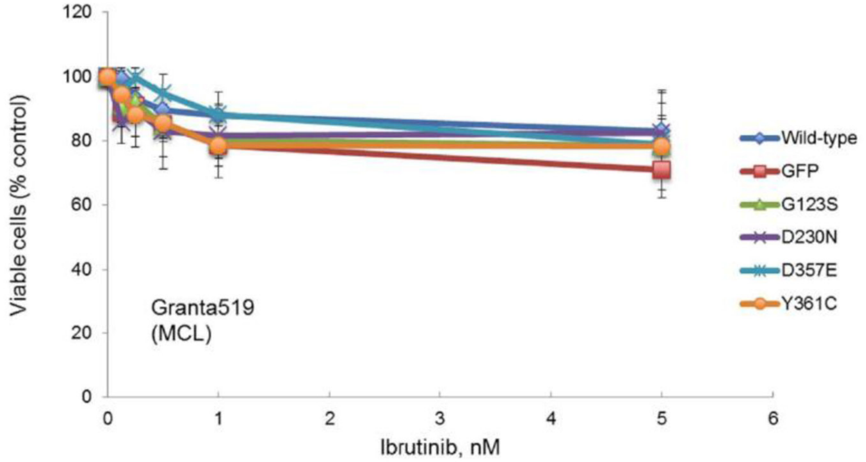

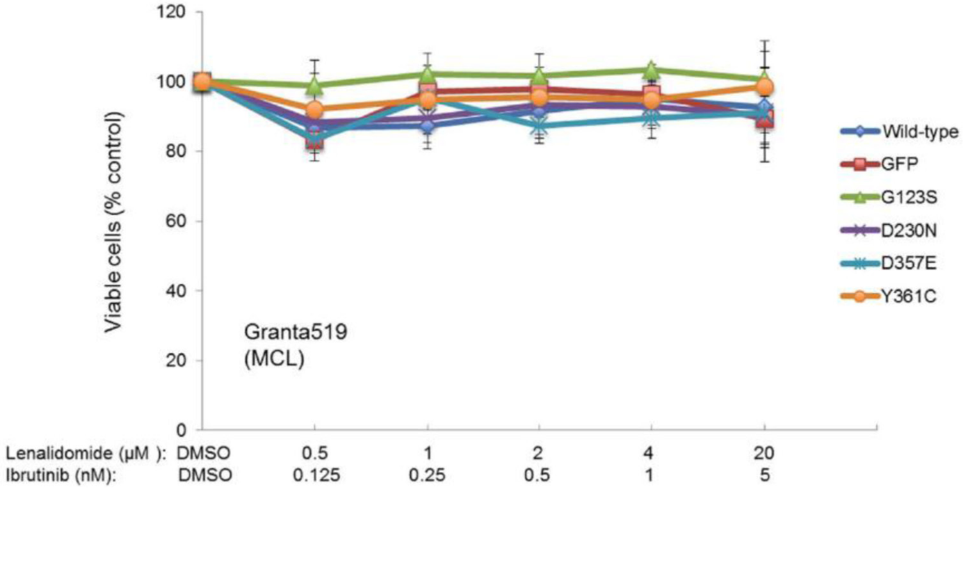

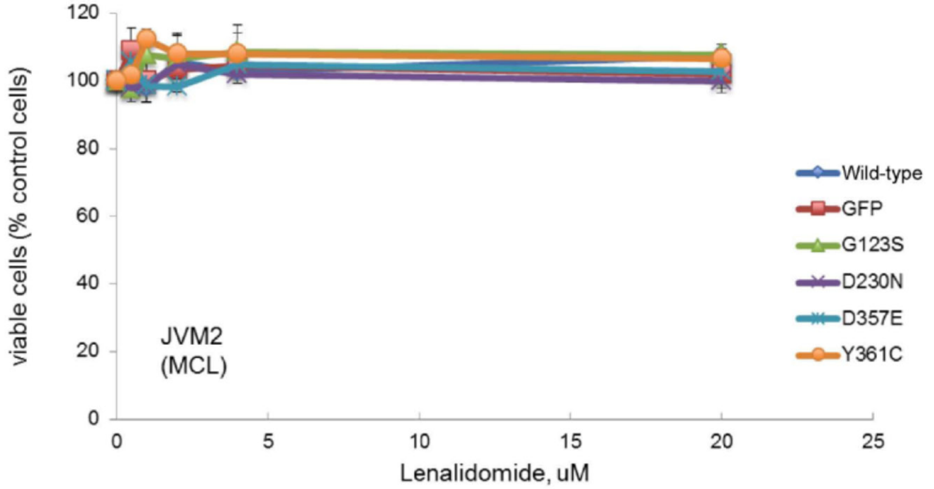

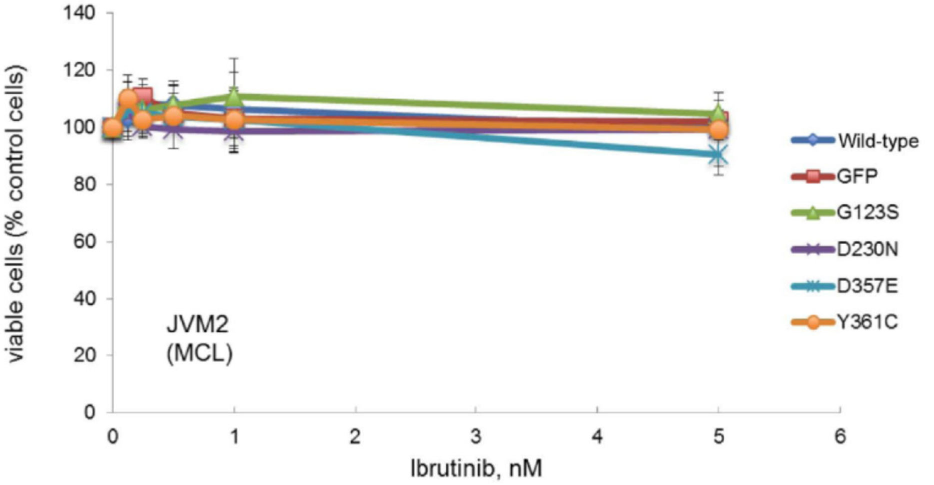

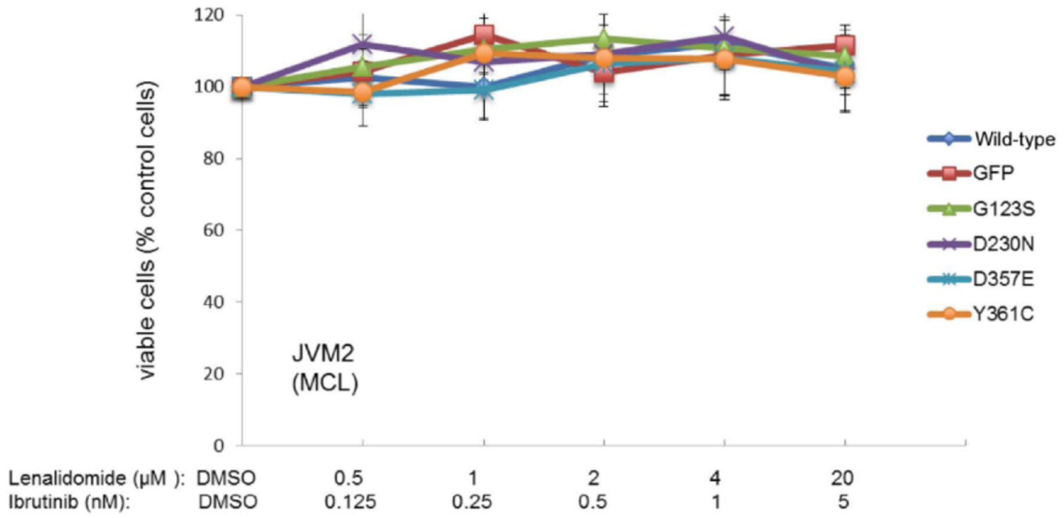

Supplementary Figure S4: Drug inhibiting assay in MCL cell lines with over expression of wild-type or mutants *CARD11* with adenovirus system. Two MCL cell lines (JVM2 and Granta519) were treated with lenalidomide, ibrutinib or with a combination of the two drugs for four days at the indicated concentrations. Error bars represent the SEM of triplicates.

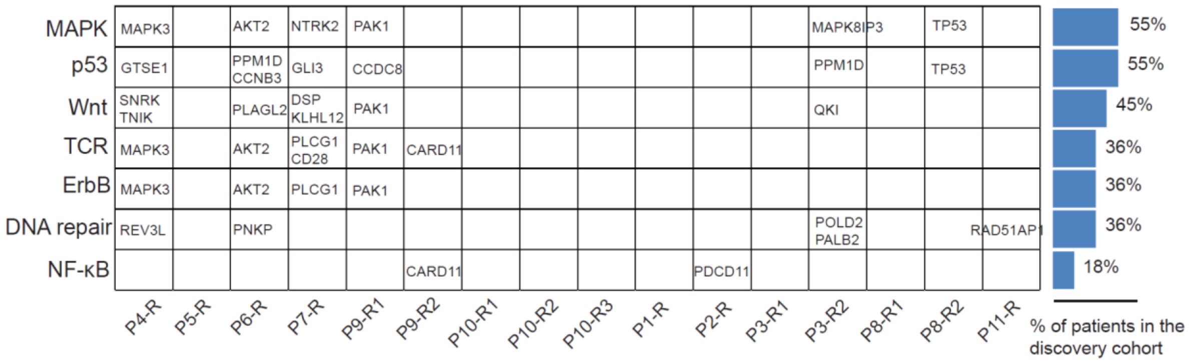

Supplementary Figure S5: Functional pathways that are recurrently affected in relapse MCLs. Genes belonging to these pathways and harboring mutations are listed. All mutated genes specific in relapse MCLs were included for pathway analysis with annotations performed by using the KEGG [2] and WebGestalt tools [3].

Supplementary Table S1: Clinical data and sample information of patients included in WES analysis

Patient ID	Tumor cell content (%)	Description of samples	Age, sex	Sample interval, (Months)	Initial treatment#	SOX11 Positive	Morphologial or histological variants	Sample original
P1-P	90	Primary	59, M		ASCT	Yes	Conventional	Lymph node
P1-R	46	Relapse		33		Yes	Conventional	Lymph node
P2-P	50	Primary	78, F		R-FC	Yes	Conventional	Bone marrow
P2-R	45	Relapse		33		Yes	Conventional	Bone marrow
P3-R1	80	Relapse 1	65, M		R-CHOP	Yes	Conventional	Lymph node
P3-R2	80	Relapse 2		33		Yes	Conventional	Lymph node
P4-P	80	Primary	77, M		CHOP, local irradiation	Yes	Conventional	Oral
P4-R	90	Relapse		64		Yes	Conventional	Pleural exsudate
P4-C*	0	Control*					Conventional	T-cell sorted from viability frozen cells
P5-P	80	Primary	82, M			Yes	Conventional	Lymph node
P5-R	80	Relapse		23		Yes	Conventional	Tonsil
P5-C*	0	Control*					Conventional	Normal control cells sorted from bone marrow
P6-P	86	Primary	56, M		ASCT	Yes	Conventional	Lymph node
P6-R	86	Relapse		97		Yes	Conventional	Intestinal
P6-C*	0	Control*					Conventional	Blood, viability frozen and tumor free
P7-P	86	Primary	52, M		ASCT	Yes	Conventional	Lymph node
P7-R	70	Relapse		123		Yes	Blastoid	Lymph node
P7-C*	0,02	Control*						Normal bone marrow sample
P8-P	66	Primary	41, M		Surgery alone	Yes	Conventional	Tonsil
P8-R1	81	Relapse 1		16	Surgery alone	Yes	Conventional	Lymph node
P8-R2	56	Relapse 2		17		Yes	Conventional	Tonsil

P9-P	80	Primary	53, M		ASCT	Yes	Conventional	Lymph node
P9-R1	80	Relapse 1		48	Rituximab	Yes	Conventional	Lymph node
P9-R2	86	Relapse 2		6		Yes	Conventional	Subcutaneous
P9-C*	0	Control*					Conventional	Blood, viability frozen and tumor free
P10-R1	63	Relapse 1	44, F		CdA	Yes	Conventional	Lymph node
P10-R2	61	Relapse 2		60	Rituximab	Yes	Conventional	Tonsil
P10-R3	82	Relapse 3		25		Yes	Conventional	Lymph node
P10-C*	0,3	Control*					Conventional	Normal bone marrow sample
P11-P	78	Primary	76, F		R-CHOP	Yes	Conventional	Bone marrow
P11-R	80	Relapse		21		Yes	Conventional	Blood
P12-P	87	Primary	41, M			Yes	Conventional	Lymph node
P12-C*	0	Control*		N.A.			Conventional	Normal bone marrow sample
P13-P	83	Primary	49, M			Yes	Conventional	Lymph node
P13-C*	0	Control*		N.A.				Normal bone marrow sample

P1, patient No.1; P, primary sample; R, relapse sample; R1, first relapse sample; R2, second relapse sample; R3, third relapse sample; C, control sample (labeled with *); N.A., not available; ASCT, Autologous Stem Cell Transplantation; R-FC, rituximab plus fludarabine, and cyclophosphamide; R-CHOP, the combination of cyclophosphamide, doxorubicin, vincristine, and prednisone (CHOP) and rituximab; CdA, cladribine; FACS, Fluorescence-activated cell sorting; #, treatment after diagnosis or at relapse as indicated.

Supplementary Table S2: Summary of the performance of exome sequencing

Sample ID	Average sequence depth	Coverage of target, %	Target covered, 10×, %	Target covered, 20×, %
**Patient samples**				
P1-P	67.1	99.2	90.7	80.7
P1-R	96.9	99.2	93.1	86.7
P2-P	76.6	96.7	85.0	75.8
P2-R	79.6	96.8	85.6	76.6
P3-R1	70.9	97.1	85.2	75.5
P3-R2	87.4	97.1	86.0	77.3
P4-P	72.9	97.8	87.5	78.1
P4-R	72.4	98.2	88.0	78.9
P4-C	88.6	98.7	89.5	81.0
P5-P	74.6	98.2	88.1	78.9
P5-R	74.9	98.0	87.2	77.6
P5-C	88.6	98.5	89.1	80.6
P6-P	76.6	98.4	88.6	80.0
P6-R	75.6	98.2	87.5	78.2
P6-C	88.6	99.0	91.1	84.1
P7-P	77.4	98.2	87.8	78.6
P7-R	42.6	97.7	81.1	65.7
P7-C	88.5	98.7	89.8	82.0
P8-P	72.1	98.0	86.8	76.9
P8-R1	61.4	98.1	87.0	76.4

P8-R2	66.0	98.1	87.1	77.0
P9-P	72.2	98.3	87.8	78.4
P9-R1	55.9	97.7	84.7	72.7
P9-R2	65.8	97.9	86.7	76.5
P9-C	88.9	97.4	82.5	70.4
P10-R1	74.4	97.9	87.5	78.2
P10-R2	69.4	97.8	87.3	77.8
P10-R3	79.3	98.1	88.3	79.5
P10-C	88.8	98.1	87.9	79.1
P11-P	75.1	98.6	90.6	83.0
P11-R	73.6	98.6	90.4	82.7
P12-P	75.2	98.9	91.2	84.0
P12-C	66.9	98.7	90.3	82.2
P13-P	126.2	99.3	94.3	89.7
P13-C	60.4	98.6	89.5	80.5
Mean	76.3	98.2	88.0	78.9
MCL cell line				
Granta519	72.9	98.7	91.1	83.4
JVM2	73.7	98.7	91.2	84.1
Rec-1	76.9	99.0	91.7	84.0
Mino	72.9	98.8	91.0	83.4
Jeko-1	64.5	98.5	89.0	79.4
Z-138	74.2	98.9	91.1	83.6
Mean	72.5	98.8	90.9	83.0

P1, patient No.1; P, primary tumor; R, relapse tumor; R1, first relapse tumor; R2, second relapse tumor; R3, third relapse tumor; C, control.

Supplementary Table S3: Somatic variants identified in 27 MCL samples from 13 patients

Supplementary Table S3A

Gene	Sample ID	Frequency at diagnosis#	Somatic or not	AA Change	Chr	Ref	Obs
ACIN1	P-1P	10%	P.S.	NM_001164814:c.T343C:p.S115P	chr14	A	G
AMBN	P-1P	11%	P.S.	NM_016519:c.G550A:p.A184T	chr4	G	A
APBB3	P-1P	10%	P.S.	NM_006051:c.C533T:p.A178V	chr5	G	A
CBS	P-1P	11%	P.S.	NM_000071:c.G619A:p.A207T	chr21	C	T
CENPJ	P-1P	22%	P.S.	NM_018451:c.G1133T:p.R378L	chr13	C	A
ERF	P-1P	11%	P.S.	NM_006494:c.A1526G:p.E509G	chr19	T	C
FANCA	P-1P	18%	P.S.	NM_000135:c.G2353A:p.A785T	chr16	C	T
GALR3	P-1P	50%	P.S.	NM_003614:c.C395T:p.A132V	chr22	C	T
HCFC1	P-1P	12%	P.S.	NM_005334:c.G437A:p.G146D	chrX	C	T
KIF15	P-1P	13%	P.S.	NM_020242:c.G11A:p.G4D	chr3	G	A
PIEZO1	P-1P	67%	P.S.	NM_001142864:c.G6905A:p. R2302H	chr16	C	T
PTGER2	P-1P	10%	P.S.	NM_000956:c.C295A:p.L99M	chr14	C	A
TBC1D3H	P-1P	11%	P.S.	NM_001123392:c.A38G:p.Q13R	chr17	T	C
TSC1	P-1P	18%	P.S.	NM_001162427:c.C2926T:p. R976W	chr9	G	A

ENO3	P-2P	13%	P.S.	NM_001193503:c.G941A:p.C314Y	chr17	G	A
CCDC18	P-4P	20%	P.S.	NM_206886:c.2705_2713del:p.902_905del	chr1	TCTCTCAAT	-
SPTBN4	P-4P	16%	somatic	NM_020971:c.A3767T:p.Q1256L	chr19	A	T
CCM2L	P-5P	10%	P.S.	NM_080625:c.G1298A:p.C433Y	chr20	G	A
PIGT	P-5P	13%	P.S.	NM_001184730:c.C1036T:p.R346W	chr20	C	T
TNPO2	P-5P	29%	somatic	NM_001136196:c.G2380A:p.A794T	chr19	C	T
ACTN2	P-6P	25%	somatic	NM_001103:c.C2147G:p.T716R	chr1	C	G
ADAM11	P-6P	18%	somatic	NM_002390:c.T1264C:p.Y422H	chr17	T	C
MYO5A	P-6P	26%	somatic	NM_000259:exon8:c.838+1G>A	chr15	C	T
POT1	P-6P	21%	somatic	NM_015450:c.G282C:p.Q94H	chr7	C	G
PPIG	P-6P	16%	somatic	NM_004792:c.G743A:p.R248Q	chr2	G	A
PRKACA	P-6P	11%	somatic	NM_002730:c.C161T:p.S54F	chr19	G	A
S1PR1	P-6P	21%	somatic	NM_001400:c.T209G:p.I70S	chr1	T	G
STK10	P-6P	19%	somatic	NM_005990:c.G178A:p.A60T	chr5	C	T
TAF7L	P-6P	31%	somatic	NM_001168474:c.T744G:p.D248E	chrX	A	C
TNR	P-6P	22%	somatic	NM_003285:c.G3668A:p.R1223H	chr1	C	T
TRIM51	P-6P	24%	somatic	NM_032681:c.G616A:p.E206K	chr11	G	A
ATM	P-7P	24%	somatic	NM_000051:c.G6188A:p.G2063E	chr11	G	A
BNC2	P-7P	15%	somatic	NM_017637:c.A223G:p.T75A	chr9	T	C
CEP152	P-7P	12%	somatic	NM_001194998:c.G1184A:p.C395Y	chr15	C	T
DNAH1	P-7P	13%	somatic	NM_015512:c.T12370G:p.L4124V	chr3	T	G
ENPP1	P-7P	11%	somatic	NM_006208:c.C1075T:p.L359F	chr6	C	T
GON4L	P-7P	11%	somatic	NM_001037533:c.C92T:p.A31V	chr1	G	A
HECTD3	P-7P	15%	somatic	NM_024602:c.C1442T:p.A481V	chr1	G	A
KIAA0895L	P-7P	11%	somatic	NM_001040715:c.C398T:p.A133V	chr16	G	A
MGAT4C	P-7P	15%	somatic	NM_013244:c.G400A:p.E134K	chr12	C	T
MSI1	P-7P	10%	somatic	NM_002442:c.C830T:p.A277V	chr12	G	A
PTPRR	P-7P	17%	somatic	NM_130846:c.G857A:p.R286Q	chr12	C	T
TBL1XR1	P-7P	10%	somatic	NM_024665:c.A1337G:p.Y446C	chr3	T	C
ZNF678	P-7P	13%	somatic	NM_178549:c.G935A:p.R312K	chr1	G	A
CD163	P-8P	26%	P.S.	NM_004244:c.G997A:p.V333I	chr12	C	T
OR10AG1	P-8P	17%	P.S.	NM_001005491:c.G783T:p.M261I	chr11	C	A
PGM2	P-8P	24%	P.S.	NM_018290:c.T1261C:p.F421L	chr4	T	C
TRPM7	P-8P	10%	P.S.	NM_017672:c.G4874C:p.R1625T	chr15	C	G
CALR	P-9P	12%	somatic	NM_004343:c.C715T:p.P239S	chr19	C	T
ACTN2	P-12P	38%	somatic	NM_001103:c.T437C:p.I146T	chr1	T	C
ATM	P-12P	75%	somatic	NM_000051:c.A6056G:p.Y2019C	chr11	A	G
BAHD1	P-12P	41%	somatic	NM_014952:c.G317T:p.S106I	chr15	G	T
CAPN7	P-12P	45%	somatic	NM_014296:c.A1424C:p.Q475P	chr3	A	C
CD33	P-12P	48%	somatic	NM_001082618:c.G688A:p.E230K	chr19	G	A
DSG1	P-12P	42%	somatic	NM_001942:c.C212A:p.A71D	chr18	C	A
DYSF	P-12P	34%	somatic	NM_001130976:c.G2494A:p.A832T	chr2	G	A
MEF2B	P-12P	37%	somatic	NM_001145785:c.A68G:p.K23R	chr19	T	C
MYOF	P-12P	44%	somatic	NM_133337:c.C2168T:p.A723V	chr10	G	A
PLOD2	P-12P	36%	somatic	NM_000935:c.A1008T:p.E336D	chr3	T	A

POM121C	P-12P	27%	P.S.	NM_001099415:c.1299delC:p.P433fs	chr7	G	-
UNC13B	P-12P	37%	somatic	NM_006377:c.G3010A:p.A1004T	chr9	G	A
VWDE	P-12P	58%	somatic	NM_001135924:c.G1834T:p.V612F	chr7	C	A
CNTN5	P-13P	36%	somatic	NM_175566:c.G1904A:p.R635H	chr11	G	A
CRYBB1	P-13P	36%	somatic	NM_001887:c.G689A:p.R230H	chr22	C	T
HNRNPA2B1	P-13P	42%	somatic	NM_002137:c.G238A:p.E80K	chr7	C	T
MAP3K14	P-13P	24%	somatic	NM_003954:c.C251G:p.A84G	chr17	G	C
NWD1	P-13P	42%	somatic	NM_001007525:c.G559A:p.V187I	chr19	G	A
PAX6	P-13P	39%	somatic	NM_001258465:c.C1193T:p.S398F	chr11	G	A
PLXNA1	P-13P	45%	somatic	NM_032242:c.G391A:p.G131S	chr3	G	A
PTPRK	P-13P	48%	somatic	NM_001135648:c.C434T:p.T145M	chr6	G	A
RSPH6A	P-13P	41%	somatic	NM_030785:c.G128A:p.R43K	chr19	C	T
SGCG	P-13P	22%	somatic	NM_000231:c.G236A:p.R79H	chr13	G	A
SRL	P-13P	15%	somatic	NM_001098814:c.C161G:p.S54C	chr16	G	C
TMEM132D	P-13P	31%	somatic	NM_133448:c.C1042T:p.R348C	chr12	G	A
USPL1	P-13P	40%	somatic	NM_005800:c.G935A:p.R312K	chr13	G	A

Variants appearing in primary MCLs (A) or relapse MCLs (B) are listed separately as well as those shared by both samples (C). Only variants with ≥10% mutated allele frequency were included.
#Frequency at diagnosis means the ratio of sequencing reads with particular mutation in primary (diagnostic) tumors. Table was sorted according to patient ID and alphanumeric order of gene name. P1, patient No.1; P, primary tumor; P.S, putatively somatic.

Supplementary Table S3B

Gene	Sample ID	Frequency at relapse#	Sanger Confirmed	Somatic or not	AA Change	Chr	Ref	Obs
CADPS2	P1-R	20%		P.S.	NM_001009571:c.C1384T:p.R462X	chr7	G	A
EVX2	P1-R	18%		P.S.	NM_001080458:c.C245T:p.T82M	chr2	G	A
FAM73A	P1-R	39%		P.S.	NM_001270384:c.G1370A:p.R457K	chr1	G	A
FUK	P1-R	11%		P.S.	NM_145059:c.A1015T:p.S339C	chr16	A	T
GCNT2	P1-R	11%		P.S.	NM_145649:c.T284G:p.F95C	chr6	T	G
GDI2	P1-R	24%		P.S.	NM_001115156:c.G32A:p.G11D	chr10	C	T
NOBOX	P1-R	31%		P.S.	NM_001080413:c.C1573T:p.P525S	chr7	G	A
OLFM3	P1-R	12%		P.S.	NM_058170:c.C325T:p.R109W	chr1	G	A
PCDHGA1	P1-R	12%		P.S.	NM_018912:c.C1976T:p.T659M	chr5	C	T
PRPF3	P1-R	18%		P.S.	NM_004698:c.G830A:p.R277H	chr1	G	A
C1orf168	P2-R	13%		P.S.	NM_001004303:c.C572T:p.A191V	chr1	G	A
CCM2L	P2-R	14%		P.S.	NM_080625:c.G1108A:p.A370T	chr20	G	A
COL6A5	P2-R	14%		P.S.	NM_001278298:c.C6671T:p.T2224M	chr3	C	T
DDOST	P2-R	14%		P.S.	NM_005216:c.T1088C:p.F363S	chr1	A	G
LPAR4	P2-R	10%		P.S.	NM_005296:c.G827A:p.R276H	chrX	G	A
LRTM2	P2-R	20%		P.S.	NM_001039029:c.C188A:p.P63H	chr12	C	A
MYCBP2	P2-R	17%	Yes	P.S.	NM_015057:c.5524_5549del:p.1842_1850del	chr13	ACTGTGGTC ATTCCTCCAT CTCCATT	-
NCAM2	P2-R	18%		P.S.	NM_004540:c.C1025G:p.T342R	chr21	C	G
NOM1	P2-R	14%		P.S.	NM_138400:c.C2225G:p.T742R	chr7	C	G
OR14C36	P2-R	18%		P.S.	NM_001001918:c.T643A:p.S215T	chr1	T	A
PDCD11	P2-R	23%	Yes	P.S.	NM_014976:c.C2021T:p.A674V	chr10	C	T

PRG4	P2-R	100%		P.S.	NM_001127710:c.1015_1038del:p.339_346del	chr1	GAGCCTG CACCCACC ACTCCCAAA	-
TM7SF2	P2-R	33%		P.S.	NM_001277233:c.G619C:p.A207P	chr11	G	C
BZW2	P3-R1	10%		P.S.	NM_001159767:c.C775T:p.Q259X	chr7	C	T
C11orf80	P3-R1	19%		P.S.	NM_024650:exon11:c.1336+1G>A	chr11	G	A
CNTNAP1	P3-R1	29%		P.S.	NM_003632:c.C1018T:p.R340W	chr17	C	T
DAPK1	P3-R1	23%		P.S.	NM_004938:c.C620A:p.T207N	chr9	C	A
FAM172A	P3-R1	16%		P.S.	NM_001163418:c.G256A:p.V86I	chr5	C	T
FHOD3	P3-R1	16%		P.S.	NM_001281739:c.C3289G:p.L1097V	chr18	C	G
FHOD3	P3-R1	21%		P.S.	NM_001281739:c.C3660G:p.F1220L	chr18	C	G
HTR4	P3-R1	29%		P.S.	NM_001040169:c.C691T:p.R231W	chr5	G	A
LDB3	P3-R1	31%		P.S.	NM_001080114:c.C1763T:p.A588V	chr10	C	T
MAP1B	P3-R1	12%		P.S.	NM_005909:c.C3388T:p.P1130S	chr5	C	T
MMD2	P3-R1	45%		P.S.	NM_001100600:c.C373T:p.L125F	chr7	G	A
MORC2	P3-R1	28%		P.S.	NM_014941:c.C2362T:p.R788W	chr22	G	A
SLC23A2	P3-R1	14%		P.S.	NM_005116:c.G754A:p.A252T	chr20	C	T
SLC4A8	P3-R1	18%		P.S.	NM_001039960:c.C1574T:p.S525F	chr12	C	T
AEBP1	P3-R2	37%		P.S.	NM_001129:c.C3440T:p.T1147I	chr7	C	T
ATF7IP	P3-R2	29%		P.S.	NM_001286514:c.A3160G:p.R1054G	chr12	A	G
BMP5	P3-R2	22%	Yes	P.S.	NM_021073:c.C595T:p.R199W	chr6	G	A
CCDC108	P3-R2	42%		P.S.	NM_001278296:c.T1690C:p.Y564H	chr2	A	G
CHSY3	P3-R2	45%		P.S.	NM_175856:c.C1675T:p.R559C	chr5	C	T
DERL1	P3-R2	18%		P.S.	NM_001134671:c.G254C:p.R85P	chr8	C	G
GIGYF2	P3-R2	60%		P.S.	NM_001103148:c.3648_3659del:p.1216_1220del	chr2	ACAGCAG CCACA	-
GPR156	P3-R2	32%		P.S.	NM_001168271:c.C1799A:p.A600E	chr3	G	T
IFT172	P3-R2	22%		P.S.	NM_015662:c.T1326G:p.S442R	chr2	A	C
IGF2BP1	P3-R2	23%	Yes	P.S.	NM_001160423:c.T1215G:p.Y405X	chr17	T	G
KIAA1199	P3-R2	11%		P.S.	NM_018689:c.C2057T:p.A686V	chr15	C	T
KIF27	P3-R2	25%		P.S.	NM_001271927:c.G397A:p.V133I	chr9	C	T
LCA5	P3-R2	29%		P.S.	NM_001122769:c.A2079T:p.E693D	chr6	T	A
LRRC8E	P3-R2	60%		P.S.	NM_001268285:c.C1132T:p.R378W	chr19	C	T
MAGEB18	P3-R2	25%		P.S.	NM_173699:c.C338T:p.S113L	chrX	C	T
MAPK8IP3	P3-R2	12%		P.S.	NM_001040439:c.C349G:p.Q117E	chr16	C	G
MARVELD2	P3-R2	18%		P.S.	NM_001244734:c.C1454T:p.S485L	chr5	C	T
NPAS4	P3-R2	18%		P.S.	NM_178864:c.G1186T:p.E396X	chr11	G	T
PALB2	P3-R2	25%	Yes	P.S.	NM_024675:c.G1139T:p.S380I	chr16	C	A
PCDHA1	P3-R2	27%		P.S.	NM_018900:c.G1868A:p.R623H	chr5	G	A
POLD2	P3-R2	39%	Yes	P.S.	NM_001127218:c.G1075A:p.E359K	chr7	C	T
PPM1D	P3-R2	10%	Yes	P.S.	NM_003620:c.G428A:p.R143H	chr17	G	A
QKI	P3-R2	34%	Yes	P.S.	NM_006775:c.A50T:p.Y17F	chr6	A	T
RASA4	P3-R2	20%		P.S.	NM_006989:c.G1915A:p.V639I	chr7	C	T
SLC35G6	P3-R2	13%		P.S.	NM_001102614:c.G458T:p.G153V	chr17	G	T
TNFRSF19	P3-R2	24%		P.S.	NM_018647:c.T1247A:p.V416E	chr13	T	A
TRIM52	P3-R2	15%		P.S.	NM_032765:c.A112T:p.N38Y	chr5	T	A
TRIOBP	P3-R2	39%		P.S.	NM_001039141:c.A1012T:p.N338Y	chr22	A	T
TTN	P3-R2	15%		P.S.	NM_003319:c.67328_67343del:p.22443_22448del	chr2	GGTCTGCCT GGTGCATCTG	-
TUBD1	P3-R2	20%		P.S.	NM_001193609:c.C508T:p.Q170X	chr17	G	A
USP1	P3-R2	21%	Yes	P.S.	NM_001017415:c.A1714G:p.T572A	chr1	A	G

ZBTB20	P3-R2	26%		P.S.	NM_001164342:c.G2113T:p.G705C	chr3	C	A
ADAMTS16	P4-R	27%		somatic	NM_139056:c.C1769T:p.S590L	chr5	C	T
CDH5	P4-R	30%		somatic	NM_001795:c.A154T:p.N52Y	chr16	A	T
COL14A1	P4-R	12%		somatic	NM_021110:c.A2248G:p.S750G	chr8	A	G
DDX17	P4-R	16%		P.S.	NM_001098504:c.2146_2149del:p.716_717del	chr22	AGGC	-
GTSE1	P4-R	10%		somatic	NM_016426:c.C554T:p.A185V	chr22	C	T
KRT73	P4-R	18%		somatic	NM_175068:c.A946G:p.K316E	chr12	T	C
MAPK3	P4-R	10%		somatic	NM_001040056:c.G484T:p.V162L	chr16	C	A
MPP1	P4-R	12%		somatic	NM_001166460:c.C233T:p.T78M	chrX	G	A
NOC3L	P4-R	31%		P.S.	NM_022451:c.828delG:p.R276fs	chr10	C	-
PCDHGA2	P4-R	16%		somatic	NM_018915:c.C1424T:p.T475M	chr5	C	T
PVRL1	P4-R	11%		P.S.	NM_002855:c.C784T:p.R262W	chr11	G	A
REV3L	P4-R	28%	Yes	somatic	NM_002912:c.C5801T:p.A1934V	chr6	G	A
RHBDF2	P4-R	29%		P.S.	NM_001005498:c.C1636T:p.P546S	chr17	G	A
SLITRK5	P4-R	13%		somatic	NM_015567:c.C1703A:p.T568N	chr13	C	A
SNRK	P4-R	14%	Yes	somatic	NM_001100594:c.G1410C:p.K470N	chr3	G	C
TNIK	P4-R	14%	Yes	somatic	NM_001161565:c.C1642T:p.R548C	chr3	G	A
TSACC	P4-R	31%		somatic	NM_144627:c.C7T:p.R3W	chr1	C	T
CLCN7	P5-R	15%		somatic	NM_001114331:c.C1762T:p.P588S	chr16	G	A
5-Sep	P6-R	10%		somatic	NM_001009939:c.G452A:p.G151D	chr22	G	A
ADI1	P6-R	14%		somatic	NM_018269:c.G433A:p.A145T	chr2	C	T
AKT2	P6-R	10%		somatic	NM_001243028:c.C322T:p.R108W	chr19	G	A
ANO1	P6-R	11%		somatic	NM_018043:c.G916A:p.A306T	chr11	G	A
AP2A1	P6-R	18%		somatic	NM_014203:c.G365C:p.S122T	chr19	G	C
BOD1L1	P6-R	43%		somatic	NM_148894:c.G8071T:p.E2691X	chr4	C	A
C14orf159	P6-R	33%		somatic	NM_001102368:c.G1007A:p.R336H	chr14	G	A
CCNB3	P6-R	12%		somatic	NM_033670:c.G331A:p.A111T	chrX	G	A
CD151	P6-R	17%		somatic	NM_001039490:c.G568A:p.A190T	chr11	G	A
COL4A1	P6-R	25%	Yes	somatic	NM_001845:c.G3059A:p.G1020E	chr13	C	T
COLEC12	P6-R	35%		somatic	NM_130386:c.C1003T:p.R335C	chr18	G	A
CPS1	P6-R	38%		somatic	NM_001875:c.C712T:p.R238X	chr2	C	T
DSG2	P6-R	43%		somatic	NM_001943:c.C1642T:p.R548C	chr18	C	T
DSG4	P6-R	36%		somatic	NM_001134453:c.G2323A:p.A775T	chr18	G	A
ETS1	P6-R	55%	Yes	somatic	NM_001162422:c.G544A:p.D182N	chr11	C	T
FBN2	P6-R	29%		somatic	NM_001999:c.G6398A:p.G2133E	chr5	C	T
GABRB3	P6-R	40%		somatic	NM_000814:c.G172T:p.G58C	chr15	C	A
HMGCL	P6-R	11%		somatic	NM_000191:c.G80A:p.G27D	chr1	C	T
HSPA9	P6-R	57%	Yes	somatic	NM_004134:c.A1000G:p.M334V	chr5	T	C
IRF5	P6-R	60%	Yes	somatic	NM_001242452:c.C634T:p.R212C	chr7	C	T
ITPR3	P6-R	13%		somatic	NM_002224:c.G6683A:p.G2228D	chr6	G	A
KATNA1	P6-R	34%		somatic	NM_007044:c.G577A:p.E193K	chr6	C	T
MDN1	P6-R	39%		somatic	NM_014611:c.A7582G:p.T2528A	chr6	T	C
MTX3	P6-R	22%		somatic	NM_001167741:c.C437T:p.A146V	chr5	G	A
MYOM2	P6-R	41%		somatic	NM_003970:c.G3008T:p.R1003L	chr8	G	T
OR5L2	P6-R	44%		somatic	NM_001004739:c.G872A:p.S291N	chr11	G	A
P4HA1	P6-R	26%		somatic	NM_000917:c.C37A:p.P13T	chr10	G	T
PAPPA2	P6-R	39%		P.S.	NM_020318:c.2324delC:p.A775fs	chr1	C	-
PCNXL2	P6-R	34%		somatic	NM_014801:c.A4215C:p.L1405F	chr1	T	G
PLAGL2	P6-R	43%	Yes	somatic	NM_002657:c.G376A:p.A126T	chr20	C	T

PNKP	P6-R	52%	Yes	somatic	NM_007254:c.C347A:p.P116Q	chr19	G	T
PPM1D	P6-R	25%	Yes	P.S.	NM_003620:c.1344delT:p.N448fs	chr17	T	-
RAD21L1	P6-R	30%		somatic	NM_001136566:c.G1326T:p.M442I	chr20	G	T
RGS9	P6-R	34%		somatic	NM_001081955:c.C286G:p.P96A	chr17	C	G
SCN10A	P6-R	27%		somatic	NM_006514:c.T728G:p.V243G	chr3	A	C
SDK1	P6-R	50%		somatic	NM_152744:c.G4180A:p.V1394M	chr7	G	A
SPI1	P6-R	33%	Yes	somatic	NM_001080547:c.C679G:p.Q227E	chr11	G	C
STK31	P6-R	26%		somatic	NM_001260504:c.G659A:p.W220X	chr7	G	A
TMEM8A	P6-R	47%		somatic	NM_021259:c.G1261A:p.V421M	chr16	C	T
TTC31	P6-R	27%		somatic	NM_022492:c.T1416G:p.H472Q	chr2	T	G
AGRP	P7-R	16%		somatic	NM_001138:c.A341G:p.N114S	chr16	T	C
B3GNT7	P7-R	35%		somatic	NM_145236:c.A968C:p.Y323S	chr2	A	C
CD28	P7-R	13%		somatic	NM_006139:c.G222C:p.Q74H	chr2	G	C
CDH9	P7-R	10%		P.S.	NM_016279:c.A1589T:p.E530V	chr5	T	A
COL3A1	P7-R	13%		somatic	NM_000090:c.C3583T:p.P1195S	chr2	C	T
COPS6	P7-R	11%		somatic	NM_006833:c.A827T:p.K276M	chr7	A	T
CRIM1	P7-R	19%		somatic	NM_016441:c.G3084T:p.Q1028H	chr2	G	T
DSP	P7-R	18%	Yes	somatic	NM_001008844:c.G3551A:p.R1184Q	chr6	G	A
GLI3	P7-R	26%	Yes	somatic	NM_000168:c.C370G:p.P124A	chr7	G	C
HOXA2	P7-R	12%		somatic	NM_006735:c.C601T:p.Q201X	chr7	G	A
ILF3	P7-R	30%		somatic	NM_001137673:c.A1631C:p.K544T	chr19	A	C
ING3	P7-R	10%		P.S.	NM_019071:c.C866T:p.A289V	chr7	C	T
KLHL12	P7-R	22%	Yes	somatic	NM_021633:c.C1638G:p.S546R	chr1	G	C
LEPREL4	P7-R	26%		somatic	NM_006455:c.G870T:p.K290N	chr17	C	A
LRRC72	P7-R	11%		somatic	NM_001195280:c.G467A:p.R156H	chr7	G	A
NTRK2	P7-R	38%		somatic	NM_001007097:c.G1241A:p.S414N	chr9	G	A
PLCG1	P7-R	19%		somatic	NM_002660:c.C1973T:p.P658L	chr20	C	T
PLCXD3	P7-R	20%		somatic	NM_001005473:c.C692T:p.A231V	chr5	G	A
SCG2	P7-R	15%		somatic	NM_003469:c.C1228G:p.L410V	chr2	G	C
ZNF808	P7-R	18%		somatic	NM_001039886:c.G1855A:p.G619R	chr19	G	A
KDM7A	P8-R1	11%		P.S.	NM_030647:c.C401T:p.A134V	chr7	G	A
LARP6	P8-R1	38%		P.S.	NM_018357:c.A917T:p.H306L	chr15	T	A
MYPN	P8-R1	25%		P.S.	NM_032578:c.C1385T:p.P462L	chr10	C	T
SMG6	P8-R1	29%		P.S.	NM_017575:c.A2306G:p.Y769C	chr17	T	C
DNAH5	P8-R2	21%		P.S.	NM_001369:c.G6748A:p.E2250K	chr5	C	T
IL22RA1	P8-R2	31%	Yes	P.S.	NM_021258:c.C1489T:p.P497S	chr1	G	A
JMJD1C	P8-R2	29%		P.S.	NM_004241:c.A3721G:p.S1241G	chr10	T	C
KRT33B	P8-R2	17%		P.S.	NM_002279:c.C896T:p.T299M	chr17	G	A
LMBR1	P8-R2	29%		P.S.	NM_022458:c.G1036C:p.V346L	chr7	C	G
PKHD1	P8-R2	11%		P.S.	NM_138694:c.G2639A:p.R880H	chr6	C	T
PSD	P8-R2	11%		P.S.	NM_002779:c.G1243A:p.A415T	chr10	C	T
TP53	P8-R2	38%	Yes	P.S.	NM_001126115:c.435_439del:p.145_147del	chr17	CAGGA	-
ADAMTS9	P9-R1	21%	Yes	somatic	NM_182920:c.G703A:p.D235N	chr3	C	T
BAI3	P9-R1	24%		somatic	NM_001704:c.C3775T:p.P1259S	chr6	C	T
CCDC41	P9-R1	18%		somatic	NM_001042399:c.T629G:p.V210G	chr12	A	C
CCDC8	P9-R1	23%	Yes	P.S.	NM_032040:c.G390T:p.M130I	chr19	C	A
CP	P9-R1	39%		somatic	NM_000096:c.G1406T:p.G469V	chr3	C	A
ENC1	P9-R1	10%		somatic	NM_001256576:c.G586T:p.A196S	chr5	C	A
FAM131A	P9-R1	19%		somatic	NM_144635:c.G16T:p.V6L	chr3	G	T

HCRTR1	P9-R1	13%		somatic	NM_001525:c.T1204A:p.L402M	chr1	T	A
MYH3	P9-R1	10%		somatic	NM_002470:c.G274A:p.A92T	chr17	C	T
OR4D10	P9-R1	15%		somatic	NM_001004705:c.C494T:p.P165L	chr11	C	T
OR5D18	P9-R1	13%		somatic	NM_001001952:c.C519A:p.N173K	chr11	C	A
PAK1	P9-R1	19%	Yes	somatic	NM_001128620:c.C1003T:p.L335F	chr11	G	A
PLS1	P9-R1	11%		somatic	NM_001145319:c.A1825C:p.K609Q	chr3	A	C
PLXNA1	P9-R1	15%		P.S.	NM_032242:c.G4795T:p.A1599S	chr3	G	T
PROM1	P9-R1	20%		somatic	NM_001145851:c.C885A:p.D295E	chr4	G	T
RNASET2	P9-R1	15%		somatic	NM_003730:c.G129T:p.W43C	chr6	C	A
RRN3	P9-R1	15%		P.S.	NM_018427:c.G440T:p.C147F	chr16	C	A
SLC12A4	P9-R1	29%		somatic	NM_001145962:c.T2930A:p.V977E	chr16	A	T
SPATA31C1	P9-R1	14%		P.S.	NM_001145124:c.G322A:p.D108N	chr9	G	A
SRBD1	P9-R1	19%		somatic	NM_018079:c.A1837T:p.M613L	chr2	T	A
YLPM1	P9-R1	31%		P.S.	NM_019589:c.339dupG:p.P113fs	chr14	-	G
ZC3H14	P9-R1	19%		somatic	NM_207662:c.C106A:p.L36I	chr14	C	A
ZC3HAV1L	P9-R1	15%		somatic	NM_080660:c.T812A:p.V271E	chr7	A	T
ZNF354C	P9-R1	19%		somatic	NM_014594:c.A435T:p.Q145H	chr5	A	T
CARD11	P9-R2	30%	Yes	somatic	NM_032415:c.G688A:p.D230N	chr7	C	T
LRRC7	P9-R2	13%		somatic	NM_020794:c.G2284A:p.D762N	chr1	G	A
GRIK2	P10-R3	15%		somatic	NM_001166247:c.G2170A:p.G724R	chr6	G	A
ABHD14B	P11-R1	12%		P.S.	NM_001254753:c.G380T:p.G127V	chr3	C	A
CCNT1	P11-R1	12%		P.S.	NM_001240:c.1658_1659insGCAA:p.N553fs	chr12	-	TTGC
FBXO28	P11-R1	10%		P.S.	NM_015176:c.C1042T:p.R348X	chr1	C	T
KLKB1	P11-R1	13%		P.S.	NM_000892:c.G1211A:p.W404X	chr4	G	A
PPRC1	P11-R1	74%		P.S.	NM_015062:c.C3575T:p.A1192V	chr10	C	T
PTPN3	P11-R1	15%		P.S.	NM_001145371:c.C250T:p.R84C	chr9	G	A
RAD51AP1	P11-R1	10%		P.S.	NM_006479:c.G751T:p.V251F	chr12	G	T
SDK2	P11-R1	11%		P.S.	NM_001144952:c.C5897T:p.S1966L	chr17	G	A

#Frequency at relapse means the ratio of sequencing reads with particular mutation in relapse tumors. Table was sorted according to patient ID and alphanumeric order of gene name. P1, patient No.1; R, relapse tumor; R1, first relapse tumor; R2, second relapse tumor; R3, third relapse tumor; P.S, putatively somatic.

Supplementary Table S3C

Gene	ID	Freq1	Freq2	Freq3	Somatic or not	Sanger confirmed	AAChange	Chr	Ref	Obs
ARAP2	P1	59%	49%		P.S.		NM_015230:c.G502T:p.D168Y	chr4	C	A
ATM	P1	71%	36%		P.S.	Yes	NM_000051:c.C9022T:p.R3008C	chr11	C	T
COL4A6	P1	100%	38%		P.S.		NM_001847:c.C2140T:p.P714S	chrX	G	A
CREBBP	P1	46%	53%		P.S.		NM_001079846:c.C2743T:p.H915Y	chr16	G	A
ENPP7	P1	38%	49%		P.S.		NM_178543:c.A998G:p.Y333C	chr17	A	G
ERBB4	P1	55%	49%		P.S.		NM_001042599:c.A1868G:p.Q623R	chr2	T	C
FAT1	P1	50%	59%		P.S.		NM_005245:c.G11003T:p.R3668L	chr4	C	A
GON4L	P1	53%	38%		P.S.		NM_001037533:c.A1315G:p.I439V	chr1	T	C
HIST1H1B	P1	37%	49%		P.S.		NM_005322:c.421_423del:p.141_141del	chr6	CTT	-
HSPD1	P1	29%	29%		P.S.		NM_002156:c.G167A:p.G56E	chr2	C	T
KMT2D	P1	45%	48%		P.S.		NM_003482:c.C12664G:p.L4222V	chr12	G	C
MPDZ	P1	50%	46%		P.S.		NM_001261406:c.T1976A:p.V659E	chr9	A	T
PTPRU	P1	52%	48%		P.S.	Yes	NM_001195001:c.C250T:p.R84X	chr1	C	T
RUNDC1	P1	35%	49%		P.S.		NM_173079:c.C823T:p.R275X	chr17	C	T

SEC14L1	P1	54%	50%		P.S.		NM_001144001:c.A528T:p.E176D	chr17	A	T
SLCO1C1	P1	35%	34%		P.S.		NM_017435:c.G308A:p.G103E	chr12	G	A
SORCS2	P1	40%	35%		P.S.		NM_020777:c.C2930A:p.T977N	chr4	C	A
SP140	P1	30%	20%		P.S.		NM_001278453:c.842_843insGTGTGATG :p.M281fs	chr2	-	GTGT GATG
STAT3	P1	56%	53%		P.S.		NM_003150:c.C1570A:p.Q524K	chr17	G	T
TENM2	P1	38%	52%		P.S.		NM_001122679:c.G2252C:p.G751A	chr5	G	C
XKR6	P1	47%	54%		P.S.		NM_173683:c.G1877A:p.R626Q	chr8	C	T
ZNF77	P1	48%	51%		P.S.		NM_021217:c.C1286T:p.T429M	chr19	G	A
ADAMTS17	P2	52%	50%		P.S.		NM_139057:c.G937A:p.E313K	chr15	C	T
ATM	P2	26%	32%		P.S.	Yes	NM_000051:c.T3431A:p.L1144X	chr11	T	A
BAZ2B	P2	42%	46%		P.S.		NM_013450:c.G4729A:p.D1577N	chr2	C	T
CHEK2	P2	31%	41%		P.S.		NM_007194:c.G1037A:p.R346H	chr22	C	T
CSMD1	P2	30%	27%		P.S.		NM_033225:c.C4546T:p.P1516S	chr8	G	A
DDX10	P2	45%	54%		P.S.		NM_004398:c.G2503A:p.D835N	chr11	G	A
EXOC6B	P2	50%	46%		P.S.		NM_015189:c.A1673G:p.Q558R	chr2	T	C
FLG	P2	51%	52%		P.S.		NM_002016:c.A9773T:p.H3258L	chr1	T	A
GRID1	P2	51%	40%		P.S.		NM_017551:c.C1150T:p.R384W	chr10	G	A
MPEG1	P2	51%	49%		P.S.		NM_001039396:c.C617T:p.T206I	chr11	G	A
MUC16	P2	53%	48%		P.S.		NM_024690:c.A37436G:p.E12479G	chr19	T	C
PDZD2	P2	41%	57%		P.S.		NM_178140:c.G1057A:p.G353R	chr5	G	A
RIMS2	P2	45%	46%		P.S.		NM_001282881:c.G1406T:p.G469V	chr8	G	T
SH3TC2	P2	32%	34%		P.S.		NM_024577:c.A904T:p.I302L	chr5	T	A
SNRNP40	P2	32%	24%		P.S.		NM_004814:c.G960T:p.L320F	chr1	C	A
SVEP1	P2	49%	52%		P.S.		NM_153366:c.A9495T:p.K3165N	chr9	T	A
TROAP	P2	20%	36%		P.S.		NM_005480:c.A1556G:p.E519G	chr12	A	G
TTN	P2	47%	42%		P.S.		NM_003319:c.C63598T:p.R21200W	chr2	G	A
UGGT2	P2	51%	42%		P.S.		NM_020121:c.G2403C:p.Q801H	chr13	C	G
USP17L2	P2	24%	24%		P.S.		NM_201402:c.C1022T:p.A341V	chr8	G	A
YLPM1	P2	57%	41%		P.S.		NM_019589:c.C1891T:p.P631S	chr14	C	T
ZBTB7B	P2	50%	50%		P.S.		NM_001256455:c.G945A:p.M315I	chr1	G	A
ZNF804A	P2	51%	45%		P.S.		NM_194250:c.A886G:p.I296V	chr2	A	G
ABCA13	P3	47%	50%		P.S.		NM_152701:c.T9361G:p.C3121G	chr7	T	G
ANKRD17	P3	50%	58%		P.S.		NM_198889:c.A7045C:p.N2349H	chr4	T	G
ASPM	P3	48%	74%		P.S.		NM_018136:c.C7312G:p.Q2438E	chr1	G	C
ATM	P3	78%	61%		P.S.	Yes	NM_000051:exon12:c.1898+1G>C	chr11	G	C
COL21A1	P3	23%	22%		P.S.		NM_030820:c.C428T:p.T143M	chr6	G	A
DENND5A	P3	49%	55%		P.S.		NM_001243254:c.T1754C:p.I585T	chr11	A	G
DMD	P3	100%	100%		P.S.		NM_004011:c.T769C:p.S257P	chrX	A	G
DNAH10	P3	36%	21%		P.S.		NM_207437:c.T4843C:p.S1615P	chr12	T	C
EP400	P3	55%	73%		P.S.		NM_015409:c.C6314T:p.P2105L	chr12	C	T
FAT1	P3	50%	44%		P.S.		NM_005245:c.C1031T:p.P344L	chr4	G	A
FLG	P3	50%	28%		P.S.		NM_002016:c.T683C:p.I228T	chr1	A	G
FLG	P3	43%	23%		P.S.		NM_002016:c.G3850T:p.E1284X	chr1	C	A
HIPK3	P3	50%	40%		P.S.		NM_001048200:c.G2657A:p.R886Q	chr11	G	A
HMCN1	P3	47%	71%		P.S.		NM_031935:c.C8764A:p.L2922I	chr1	C	A
MYO10	P3	61%	63%		P.S.		NM_012334:c.A4075G:p.N1359D	chr5	T	C
PCLO	P3	21%	18%		P.S.		NM_014510:c.C10849T:p.P3617S	chr7	G	A
PTPN14	P3	48%	77%		P.S.		NM_005401:c.A700G:p.I234V	chr1	T	C
RIMS2	P3	46%	33%		P.S.		NM_001282881:c.C2222T:p.S741L	chr8	C	T

RPGRIP1L	P3	51%	47%		P.S.		NM_001127897:c.T2009G:p.L670W	chr16	A	C
ZNF335	P3	45%	50%		P.S.		NM_022095:c.C2131T:p.R711C	chr20	G	A
ANKS1A	P4	44%	28%		somatic		NM_015245:c.A3309T:p.E1103D	chr6	A	T
AP2M1	P4	27%	48%		somatic		NM_001025205:c.G373A:p.E125K	chr3	G	A
ASB4	P4	44%	55%		somatic		NM_016116:c.G862A:p.A288T	chr7	G	A
ATM	P4	73%	98%		somatic		NM_000051:c.G9023A:p.R3008H	chr11	G	A
CABP2	P4	46%	52%		somatic		NM_016366:c.A325T:p.T109S	chr11	T	A
CHSY3	P4	45%	59%		somatic		NM_175856:c.G1253A:p.R418H	chr5	G	A
DGKG	P4	36%	34%		somatic		NM_001080744:c.940delG:p.V314fs	chr3	C	-
DGKI	P4	30%	48%		somatic		NM_004717:c.G2914A:p.G972R	chr7	C	T
DSCAM	P4	33%	49%		somatic		NM_001271534:c.G3860A:p.R1287Q	chr21	C	T
FAM81B	P4	46%	68%		somatic		NM_152548:c.C367T:p.R123C	chr5	C	T
FAT1	P4	36%	56%		somatic		NM_005245:c.C1882T:p.R628X	chr4	G	A
FOXRED1	P4	38%	30%		somatic		NM_017547:c.G1418A:p.R473H	chr11	G	A
LEMD3	P4	25%	48%		somatic		NM_001167614:c.A1760T:p.D587V	chr12	A	T
MAPRE3	P4	28%	32%		somatic		NM_012326:c.536dupC:p.A179fs	chr2	-	C
MED13	P4	30%	41%		somatic		NM_005121:c.T566G:p.L189R	chr17	A	C
NOLC1	P4	34%	43%		somatic		NM_001284388:c.G1618T:p.E540X	chr10	G	T
NRG1	P4	30%	53%		somatic		NM_001159996:c.C382T:p.L128F	chr8	C	T
PCDHGA5	P4	34%	42%		somatic		NM_018918:c.G779A:p.R260Q	chr5	G	A
PPIG	P4	36%	56%		somatic		NM_004792:c.T206A:p.V69D	chr2	T	A
PPP2R4	P4	34%	54%		somatic		NM_001271832:c.C587T:p.S196L	chr9	C	T
PRKD3	P4	34%	53%		somatic		NM_005813:c.G2492A:p.W831X	chr2	C	T
SHROOM3	P4	38%	46%		somatic		NM_020859:c.G1565A:p.G522E	chr4	G	A
SMAD2	P4	56%	74%		somatic		NM_001135937:c.T1239A:p.N413K	chr18	A	T
TAF1L	P4	39%	44%		somatic		NM_153809:c.C778T:p.R260C	chr9	G	A
TBC1D5	P4	40%	27%		somatic		NM_001134380:c.G450A:p.W150X	chr3	C	T
TMEM67	P4	37%	34%		somatic		NM_153704:c.G2924A:p.R975H	chr8	G	A
UBR5	P4	37%	46%		somatic		NM_001282873:c.C5810G:p.P1937R	chr8	G	C
ZFHX3	P4	27%	37%		somatic		NM_006885:c.G2422T:p.V808L	chr16	C	A
ANKRD30A	P5	22%	17%		somatic		NM_052997:c.C1721A:p.T574N	chr10	C	A
ARHGAP32	P5	45%	53%		somatic		NM_001142685:c.A869G:p.D290G	chr11	T	C
ARHGAP6	P5	87%	80%		somatic		NM_006125:c.C1126T:p.R376X	chrX	G	A
ATM	P5	90%	95%		somatic		NM_000051:c.G9023A:p.R3008H	chr11	G	A
BRINP3	P5	22%	15%		somatic		NM_199051:c.G1396A:p.G466S	chr1	C	T
CA3	P5	57%	58%		somatic		NM_005181:c.C709T:p.P237S	chr8	C	T
CACNA1I	P5	52%	46%		somatic		NM_001003406:c.G472A:p.V158I	chr22	G	A
CARD11	P5	13%	10%		somatic		NM_032415:c.A644T:p.K215M	chr7	T	A
CHD2	P5	28%	30%		somatic		NM_001271:c.2293dupT:p.H764fs	chr15	-	T
CPO	P5	26%	21%		somatic		NM_173077:c.T578A:p.I193N	chr2	T	A
DGKB	P5	15%	13%		somatic		NM_004080:exon14:c.1170+2T>C	chr7	A	G
DNAH8	P5	14%	21%		somatic		NM_001206927:c.C9535T:p.R3179C	chr6	C	T
FRMD6	P5	44%	48%		somatic		NM_001267046:c.G335A:p.R112H	chr14	G	A
GPR32	P5	46%	55%		somatic		NM_001506:c.G235A:p.V79I	chr19	G	A
IQCF3	P5	25%	53%		somatic		NM_001085479:c.G236A:p.R79Q	chr3	G	A
ITIH3	P5	38%	26%		somatic		NM_002217:c.C2032T:p.R678C	chr3	C	T
KCNU1	P5	20%	18%		somatic		NM_001031836:c.A2228T:p.Y743F	chr8	A	T
KIAA1549	P5	38%	43%		somatic		NM_001164665:c.C5716T:p.P1906S	chr7	G	A
KLHL1	P5	54%	50%		somatic		NM_020866:c.C2164A:p.P722T	chr13	G	T
LAMP3	P5	53%	52%		somatic		NM_014398:c.T1118C:p.V373A	chr3	A	G

LZTS1	P5	52%	71%		somatic		NM_021020:c.G1523A:p.R508Q	chr8	C	T
MSTN	P5	38%	48%		somatic		NM_005259:c.G1112A:p.R371H	chr2	C	T
NID1	P5	45%	40%		somatic		NM_002508:c.G905C:p.R302P	chr1	C	G
PITPNM1	P5	33%	67%		somatic		NM_001130848:c.C2199A:p.D733E	chr11	G	T
PNLDC1	P5	15%	19%		somatic		NM_001271862:c.T503A:p.V168E	chr6	T	A
RB1CC1	P5	49%	45%		somatic		NM_001083617:c.G2659A:p.E887K	chr8	C	T
SEMG2	P5	17%	13%		somatic		NM_003008:c.C278T:p.A93V	chr20	C	T
UNC79	P5	24%	15%		somatic		NM_020818:c.C7346T:p.S2449L	chr14	C	T
MEF2B	P6	19%	33%		somatic		NM_001145785:c.A146G:p.N49S	chr19	T	C
SIX4	P6	32%	44%		somatic		NM_017420:c.T994A:p.Y332N	chr14	A	T
SORL1	P6	23%	32%		somatic		NM_003105:c.C543G:p.D181E	chr11	C	G
ALPK2	P7	33%	47%		somatic		NM_052947:c.A405C:p.E135D	chr18	T	G
ARHGAP32	P7	47%	60%		somatic		NM_014715:c.C2051T:p.P684L	chr11	G	A
ATM	P7	38%	46%		somatic		NM_000051:c.7761dupG:p.V2587fs	chr11	-	G
BTBD11	P7	13%	13%		somatic		NM_001017523:c.C1235T:p.A412V	chr12	C	T
FAM184B	P7	30%	26%		somatic		NM_015688:c.A1031G:p.D344G	chr4	T	C
FOXO1	P7	18%	27%		somatic		NM_002015:c.A1100G:p.N367S	chr13	T	C
HNRNPK	P7	37%	32%		somatic		NM_031263:exon6:c.213+1G>A	chr9	C	T
KCNH1	P7	45%	58%		somatic		NM_002238:c.C1939T:p.R647W	chr1	G	A
LRP1	P7	32%	48%		somatic		NM_002332:c.9979_9989del:p.3327_3330del	chr12	GGGCGCACCTG	-
SMARCA4	P7	46%	67%		somatic		NM_001128845:c.G2371A:p.A791T	chr19	G	A
SRF	P7	16%	39%		somatic		NM_003131:c.G844A:p.A282T	chr6	G	A
SYTL2	P7	29%	41%		somatic		NM_206927:c.131dupA:p.N44fs	chr11	-	T
TLR10	P7	29%	46%		somatic		NM_001017388:c.T2045C:p.I682T	chr4	A	G
UGT2B17	P7	29%	33%		somatic		NM_001077:c.G394A:p.V132I	chr4	C	T
UNC13C	P7	54%	39%		somatic		NM_001080534:c.G1078T:p.A360S	chr15	G	T
AHNAK2	P8	100%	100%	100%	P.S.		NM_138420:c.C6133G:p.L2045V	chr14	G	C
DGKB	P8	39%	58%	45%	P.S.		NM_004080:c.C488T:p.T163M	chr7	G	A
DIAPH3	P8	53%	52%	53%	P.S.		NM_030932:c.G2336A:p.R779H	chr13	C	T
FAT1	P8	46%	53%	57%	P.S.		NM_005245:c.G2734A:p.V912I	chr4	C	T
FAT4	P8	41%	51%	52%	P.S.		NM_024582:c.C13201T:p.P4401S	chr4	C	T
GPR98	P8	52%	52%	42%	P.S.		NM_032119:c.A12818G:p.H4273R	chr5	A	G
HYDIN	P8	21%	35%	28%	P.S.		NM_001270974:c.C8971A:p.P2991T	chr16	G	T
ITGAV	P8	52%	49%	46%	P.S.		NM_001145000:c.A1837G:p.I613V	chr2	A	G
KMT2D	P8	24%	32%	28%	P.S.		NM_003482:c.C4895A:p.S1632X	chr12	G	T
MUC2	P8	63%	62%	53%	P.S.		NM_002457:c.C6005T:p.T2002M	chr11	C	T
NIN	P8	37%	38%	63%	P.S.		NM_016350:c.G1549A:p.G517S	chr14	C	T
PROS1	P8	50%	56%	45%	P.S.		NM_000313:c.T1095G:p.N365K	chr3	A	C
SCN7A	P8	47%	48%	55%	P.S.		NM_002976:c.G1800A:p.M600I	chr2	C	T
SETD2	P8	53%	56%	50%	P.S.		NM_014159:c.G3251A:p.S1084N	chr3	C	T
XIRP2	P8	44%	54%	43%	P.S.		NM_001199144:c.C4502T:p.S1501F	chr2	C	T
ZNF296	P8	15%	33%	31%	P.S.		NM_145288:c.575dupC:p.P192fs	chr19	-	G
ZNF729	P8	51%	47%	43%	P.S.		NM_001242680:c.1769_1770del:p.590_590del	chr19	AT	-
ACSM5	P9	33%	47%	32%	somatic		NM_017888:c.C113T:p.T38I	chr16	C	T
ANKFN1	P9	35%	43%	46%	somatic		NM_153228:c.G2188A:p.V730I	chr17	G	A
ARF3	P9	43%	32%	45%	somatic		NM_001659:c.A400G:p.M134V	chr12	T	C
CERS3	P9	30%	53%	19%	somatic		NM_178842:c.C331T:p.R111C	chr15	G	A
CHMP3	P9	50%	49%	53%	somatic		NM_001005753:c.208_210del:p.70_70del	chr2	CTT	-

DUOX2	P9	47%	37%	50%	somatic		NM_014080:c.G3391A:p.A1131T	chr15	C	T
GAREM	P9	41%	47%	52%	somatic		NM_001242409:c.G2074A:p.V692I	chr18	C	T
MEF2B	P9	45%	48%	60%	somatic		NM_001145785:c.A68G:p.K23R	chr19	T	C
NRF1	P9	49%	52%	42%	somatic		NM_001040110:c.C730T:p.R244W	chr7	C	T
PCDHA11	P9	51%	47%	52%	somatic		NM_018902:c.G1372A:p.E458K	chr5	G	A
PCDHB6	P9	48%	52%	50%	somatic		NM_018939:c.C1217T:p.A406V	chr5	C	T
PCLO	P9	46%	44%	43%	somatic		NM_014510:c.C10943A:p.P3648H	chr7	G	T
S1PR1	P9	33%	48%	31%	somatic		NM_001400:c.364dupG:p.E121fs	chr1	-	G
SLITRK5	P9	58%	47%	39%	somatic		NM_015567:c.G1204A:p.A402T	chr13	G	A
SPOCK3	P9	47%	43%	39%	somatic		NM_001251967:c.G437C:p.S146T	chr4	C	G
TMED3	P9	43%	54%	38%	somatic		NM_007364:c.C266T:p.T89M	chr15	C	T
TNP1	P9	44%	57%	69%	somatic		NM_003284:c.G38A:p.R13K	chr2	C	T
TRIM23	P9	44%	54%	39%	somatic		NM_001656:c.C217G:p.P73A	chr5	G	C
UNC45B	P9	49%	48%	41%	somatic		NM_001033576:c.G44A:p.R15Q	chr17	G	A
WHSC1	P9	42%	48%	39%	somatic		NM_001042424:c.A3448G:p.T1150A	chr4	A	G
ZFHX4	P9	47%	49%	44%	somatic		NM_024721:c.A10501G:p.T3501A	chr8	A	G
ADCK1	P10	11%	18%	19%	somatic		NM_001142545:c.G1307T:p.G436V	chr14	G	T
ATM	P10	33%	33%	33%	somatic		NM_000051:c.C7466G:p.S2489C	chr11	C	G
BIRC3	P10	21%	34%	33%	somatic		NM_001165:c.1754_1757del:p.585_586del	chr11	TAAG	-
C7orf62	P10	23%	21%	29%	somatic		NM_152706:c.C298T:p.L100F	chr7	G	A
CASR	P10	27%	28%	28%	somatic		NM_000388:c.C2549T:p.A850V	chr3	C	T
CTNND2	P10	19%	24%	25%	somatic		NM_001332:c.C1943T:p.T648M	chr5	G	A
GPR126	P10	27%	20%	25%	somatic		NM_001032394:c.C2888T:p.A963V	chr6	C	T
LGSN	P10	17%	21%	21%	somatic		NM_016571:c.G649A:p.E217K	chr6	C	T
NXPH2	P10	20%	26%	19%	somatic		NM_007226:c.A656G:p.H219R	chr2	T	C
OR10H4	P10	18%	27%	24%	somatic		NM_001004465:c.G820A:p.A274T	chr19	G	A
PAPPA	P10	26%	26%	20%	somatic		NM_002581:c.G2669A:p.R890Q	chr9	G	A
PDE6C	P10	20%	19%	24%	somatic		NM_006204:c.C1528T:p.R510C	chr10	C	T
PKHD1	P10	18%	20%	12%	somatic		NM_138694:c.G8672A:p.R2891H	chr6	C	T
SCN11A	P10	20%	21%	16%	somatic			chr3	C	T
TEAD1	P10	25%	24%	27%	somatic		NM_021961:c.G779A:p.R260H	chr11	G	A
TTN	P10	33%	22%	26%	somatic		NM_003319:c.G4025T:p.G1342V	chr2	C	A
UNC80	P10	15%	21%	22%	somatic		NM_032504:c.T5944A:p.C1982S	chr2	T	A
ZNF615	P10	24%	20%	15%	somatic		NM_198480:c.G340A:p.V114I	chr19	C	T
ABCA13	P11	53%	54%		P.S.		NM_152701:c.T2309C:p.I770T	chr7	T	C
ABCA3	P11	41%	35%		P.S.		NM_001089:c.G754A:p.A252T	chr16	C	T
ANKRD17	P11	24%	41%		P.S.		NM_032217:c.303_314del:p.101_105del	chr4	CCGCCT CCACCG	-
BPTF	P11	93%	87%		P.S.		NM_004459:c.541_543del:p.181_181del	chr17	GAC	-
CIITA	P11	35%	41%		P.S.		NM_000246:c.A2059G:p.T687A	chr16	A	G
DCDC5	P11	49%	42%		P.S.		NM_020869:c.T1708A:p.F570I	chr11	A	T
DNAH5	P11	58%	46%		P.S.		NM_001369:c.G6748A:p.E2250K	chr5	C	T
DNAH9	P11	47%	47%		P.S.		NM_004662:c.A362C:p.E121A	chr17	A	C
FAT3	P11	40%	33%		P.S.		NM_001008781:c.G3286A:p.E1096K	chr11	G	A
FMO2	P11	42%	45%		P.S.		NM_001460:c.T928G:p.S310A	chr1	T	G
GREB1	P11	65%	62%		P.S.		NM_014668:c.G3424A:p.G1142S	chr2	G	A
GREB1	P11	61%	64%		P.S.		NM_014668:c.C3515T:p.A1172V	chr2	C	T
GRIP2	P11	25%	21%		P.S.		NM_001080423:c.C394T:p.R132C	chr3	G	A
KMT2D	P11	32%	29%		P.S.		NM_003482:c.968_969insGTGTG:p. C323fs	chr12	-	CACAC

LTN1	P11	55%	39%		P.S.		NM_015565:c.C5012T:p.T1671M	chr21	G	A
MACF1	P11	51%	42%		P.S.		NM_012090:c.A14530G:p.T4844A	chr1	A	G
MEF2B	P11	49%	37%		P.S.		NM_001145785:c.A68G:p.K23R	chr19	T	C
RYR2	P11	48%	64%		P.S.		NM_001035:c.T8162C:p.I2721T	chr1	T	C
ZNF142	P11	47%	50%		P.S.		NM_001105537:c.C3502G:p.R1168G	chr2	G	C

Table was sorted according to patient ID and alphanumeric order of gene name. Freq1: the allele frequency of mutation found in primary MCL (or in first relapse samples for P3, P10); Freq2: the allele frequency of mutation found in first relapse MCL (or in second relapse samples for P3, P10); Freq3: the allele frequency of mutation found in second relapse MCL (or in third relapse sample for P10); P.S, putatively somatic.

Supplementary Table S4: List of recurrently mutated genes reported in recent MCL and DLBCL literatures

Gene mutated	Ref.	Gene mutated	Ref.	Gene mutated	Ref.	Gene mutated	Ref.	Gene mutated	Ref.	Gene mutated	Ref.
A2ML1	5	*CDS1*	3	*FLRT2*	3	*LRRC7*	3	*PDK4*	3	*SPEF2*	3
AADACL3	3	*CECR1*	3,4	*FLT1*	5	*LRRIQ1*	3	*PDLIM3*	8	*SPEG*	4
ABCA13	3	*CELSR2*	4	*FMO2*	4	*LRRIQ3*	3	*PDLIM4*	3	*SPEN*	3,5
ABCA3	4,8	*CEP70*	3	*FRAS1*	3	*LRRN3*	3,4,5	*PDPN*	3	*SPESP1*	5
ABCA4	3,4	*CHD2*	7	*FREM1*	3	*LTB*	3	*PDS5A*	3	*SPG20*	3
ABCA9	3	*CHD3*	4,5	*FREM2*	3	*LTBP2*	5	*PDS5B*	3	*SPHKAP*	3
ABCB1	5	*CHD4*	3	*FREM2*	5	*LTN1*	5	*PDZD2*	3	*SPNS1*	3
ABCC12	5	*CHEK1*	7	*FRMPD1*	4	*LUZP1*	3	*PDZRN3*	4	*SPTA1*	3
ABCC6	3	*CHEK2*	7	*FRYL*	5	*LUZP4*	8	*PDZRN4*	4	*SPTBN1*	3,4
ABCC9	3,8	*CHMP4C*	8	*GABBR1*	3	*LYN*	5	*PEG3*	5	*SPTBN2*	5
AC092142.5	4	*CHRM2*	3	*GABRA1*	3,5	*LYST*	4	*PFAS*	5	*SPTY2D1*	5
AC121493.1	4	*CHRM3*	5	*GABRA2*	3	*MACF1*	3	*PFN1*	3	*SRC*	3
ACE	4	*CHRM5*	4	*GABRG1*	3,4	*MAGEB16*	3	*PGM2L1*	5	*SRCAP*	3
ACHE	5	*CIC*	3,4	*GABRG2*	3	*MAGEC1*	3	*PGR*	3	*SRP54*	5
ACSF3	3	*CIITA*	3,5	*GABRQ*	3	*MAGEC3*	1,2	*PHACTR4*	3	*SRPX*	3
ACSM2B	3	*CLASP1*	5	*GALNT9*	3	*MAGEL2*	3	*PHF23*	3	*SSH1*	3
ACSM3	4	*CLCA4*	3	*GAS2L1*	3	*MAGI1*	3	*PHIP*	3	*SSPO*	3
ACSS2	4	*CLEC16A*	3	*GBA3*	3	*MAGI2*	3	*PHKA2*	3	*ST8SIA3*	4
ACSS3	4	*CNGA4*	4	*GCN1L1*	4	*MAGI3*	3	*PHOX2B*	3	*STAG3*	3
ACTB	3,5	*CNOT6*	4	*GJA8*	4	*MAN2A2*	3	*PICALM*	3	*STAT3*	3,5
ACTG1	5	*CNP*	4	*GJB4*	4	*MAP2*	3	*PIK3C2G*	3	*STAT6*	3
ACTN1	4	*CNTD1*	3	*GLI2*	3	*MAP2K1*	3	*PIK3CD*	4	*STOML3*	3
ADAM2	3	*CNTLN*	3	*GLI3*	3	*MARCH7*	4	*PIK3R1*	4	*SUGT1*	5
ADAM2	4	*CNTN3*	3	*GNA13*	3,4	*MARK2*	5	*PIM1*	1,2,3,4,5	*SULF2*	4
ADAMTS1	3	*CNTN6*	4	*GNAI2*	3,4	*MBL2*	4	*PIP4K2A*	3	*SULT1C3*	3
ADAMTS12	3	*COBL*	3	*GNAT3*	5	*MBP*	3	*PIWIL1*	3	*SUPT16H*	3
ADAMTS15	4	*COBL*	5	*GNPDA1*	3	*MCC*	3	*PKD1*	3	*SV2A*	5
ADAMTS16	3	*COBLL1*	3	*GNPDA2*	3	*MCF2*	3	*PKHD1*	5	*SVEP1*	3,5
ADAMTS17	3	*COG8*	3	*GON4L*	5	*MCF2L*	3	*PLA2G4A*	3	*SVIL*	3
ADAMTS5	3	*COL11A1*	3,9	*GOPC*	4	*MDGA2*	5	*PLA2G4B*	4	*SYK*	3
ADAMTS9	3,5	*COL12A1*	3	*GP6*	3	*ME3*	3	*PLEC*	3	*SYMPK*	3
ADAMTSL1	5	*COL14A1*	3	*GPC3*	3	*MECOM*	3	*PLEKHA5*	3	*SYN2*	3
ADAMTSL3	1,2	*COL16A1*	9	*GPCPD1*	5	*MED12L*	1,2	*PLEKHA7*	4	*SYNE1*	3,5,8
ADCY2	3	*COL21A1*	5	*GPD2*	4	*MED13L*	4	*PLS1*	5	*SYNPO2*	3
ADCY5	3	*COL22A1*	3,5	*GPR133*	3,4	*MED26*	3	*PLXNA2*	3	*SYT3*	3

ADH7	4	*COL24A1*	3	*GPR149*	3	*MEF2B*	1	*PLXNA3*	3	*TACC2*	3
AFAP1L2	4	*COL4A3*	3	*GPR15*	4	*MEF2B*	2,3,4, 7,8	*PLXNB2*	3	*TAF1*	5
AGXT2L1	4	*COL4A4*	3	*GPR37*	5	*MEF2C*	3	*PLXNB3*	3,9	*TAF1L*	3,4
AHNAK	3	*COL4A5*	5	*GPR64*	5	*MEP1A*	3	*PMS1*	1,2	*TAF4B*	4
AHNAK2	3	*COL4A6*	3	*GPR98*	3	*MFHAS1*	5	*PNLIPRP1*	4	*TAOK1*	3
AHR	4	*COL5A2*	4	*GRB2*	3,5	*MGA*	3	*PNLIPRP3*	3	*TATDN3*	3
AKAP6	3	*COLEC12*	3	*GREB1*	3	*MGAM*	3	*PNPT1*	4	*TBC1D26*	9
AKAP8	1,2	*CORO2A*	4	*GREM2*	4	*MIA3*	3	*POGZ*	3	*TBC1D8B*	3,5
AKAP9	4,5	*CORO7*	4	*GRIA2*	3,4	*MIB1*	4	*POLE*	4	*TBL1XR1*	3,5
ALAS1	4	*CPS1*	3,5	*GRIA3*	3	*MICAL2*	3	*POLR1B*	3	*TBP*	3
ALDH1L2	4	*CPSF6*	3	*GRID1*	3	*MICAL3*	3	*POSTN*	5	*TCF4*	3
ALG13	5	*CPXM2*	3	*GRIK2*	3	*MKRN3*	3	*POT1*	9	*TCHH*	3,5
ALPK3	3	*CRB1*	3	*GRIN2A*	9	*MLL*	3	*POU2F2*	3,4	*TDRD1*	3
AMAC1	4	*CREBBP*	1,2,3, 4,5	*GRIN2B*	3	*MLL2*	1,2,3, 5,8,9	*PPARGC1A*	4	*TDRD5*	3
AMIGO3	4	*CRELD2*	4	*GRIP2*	3	*MLL3*	3,4,5,9	*PPEF1*	3	*TDRD6*	5
AMPH	4	*CRISPLD1*	3	*GRM2*	3	*MLL5*	3	*PPFIA2*	5	*TET2*	7
ANAPC5	3	*CRTAC1*	3	*GRM5*	4	*MLPH*	3	*PPP1R16B*	5	*TGFBR3*	5
ANGPT1	3	*CRTC3*	4	*GRM7*	3	*MMP1*	3	*PPP1R9A*	5	*THBS4*	4
ANK2	4,9	*CRY1*	3	*GRM8*	5	*MN1*	4	*PPWD1*	4	*THSD4*	3
ANK3	3	*CRYBG3*	8	*H1FOO*	3	*MORC2*	4	*PRDM1*	1,2,5	*THSD7A*	3
ANK3	5	*CSMD1*	3,5	*H6PD*	4	*MPDZ*	5	*PRDM15*	5	*THSD7B*	3
ANKHD1-EIF4EBP3	3	*CSMD2*	5,8	*HACE1*	4	*MPEG1*	5	*PRDM16*	4	*TIAM1*	3
ANKLE2	1,2	*CSMD3*	3,8	*HAP1*	3	*MPL*	4	*PRDM9*	3	*TIMM50*	4
ANKRD11	5	*CSNK2A1*	3	*HAS2*	3	*MRGPRF*	9	*PREX2*	3	*TJP1*	3
ANKRD12	3	*CSPG4*	3	*HCK*	4	*MSH3*	3	*PRKCB*	3	*TLL2*	1,2
ANKRD17	4	*CST7*	4	*HEATR3*	3	*MSH6*	5	*PRKCB1*	4	*TLN1*	5
ANKRD30A	3	*CTNNA2*	3,9	*HECW1*	5	*MSR1*	3	*PRKCD*	5	*TLN2*	4
ANKRD44	4	*CTNNA3*	4	*HELT*	3	*MTF2*	3	*PRKCQ*	4	*TLR2*	8
ANKRD5	3	*CTNNAL1*	3	*HELZ*	3	*MTL5*	5	*PRKDC*	3	*TLR7*	3
ANKRD50	3,5	*CTSZ*	3	*HEPH*	3,9	*MTMR3*	4	*PROM2*	5	*TMC1*	4
ANO5	3	*CUBN*	3	*HERC1*	3	*MTMR8*	1,2	*PROS1*	4	*TMEM132B*	3
ANTXR1	4	*CUL1*	7	*HIPK3*	4	*MTOR*	4	*PRRC2B*	5	*TMEM161A*	4
AP002358.3	4	*CUL4B*	5	*HIST1H1B*	3	*MUC16*	3,5	*PRSS38*	3	*TMEM16E*	4
AP1G2	4	*CWH43*	3	*HIST1H1C*	3,4,	*MUC2*	3	*PRSS7*	4	*TMEM2*	3
AP3B1	3,4	*CXCL5*	5	*HIST1H1D*	5	*MUC4*	3	*PSMD2*	3	*TMEM30A*	1,2,3,4
APC	4	*CXCR4*	5	*HIST1H1E*	3,4,5	*MUC5B*	3,5	*PTCHD2*	3	*TMEM49*	3
APC2	3	*CXCR5*	3	*HIST1H2AC*	5	*MYBPHL*	5	*PTGIS*	3	*TMEM63A*	4
APOA5	4	*CXorf22*	3	*HIST1H2AL*	3	*MYC*	1,2,3	*PTPN14*	4	*TMSB4X*	5
APOBEC2	3	*CXorf57*	5	*HIST1H2AM*	3,5	*MYD88*	1,2,3, 4,5	*PTPN6*	3	*TMSL3*	3
APP	4	*CYFIP1*	3	*HIST1H2BC*	3	*MYH15*	3,5	*PTPRB*	3	*TNF*	3
ARAP2	5	*CYP24A1*	4,5	*HIST1H2BK*	3,4	*MYH3*	3	*PTPRCAP*	7	*TNFAIP3*	1,2,4,5
ARHGAP20	3	*CYP2C18*	4	*HIST1H2BN*	5	*MYH7*	3	*PTPRD*	3	*TNFRSF14*	3,5
ARHGAP25	3	*CYP4B1*	3	*HIST1H2BO*	3	*MYH7B*	5	*PTPRF*	4	*TNFSF13B*	5
ARHGAP32	3	*DAAM2*	3	*HIST1H3B*	3,4	*MYH8*	3	*PTPRM*	3	*TNFSF14*	4
ARHGAP36	3	*DAPK1*	3	*HIST1H3C*	3,5	*MYL7*	4	*PTPRS*	5	*TNNI3K*	3
ARHGAP6	3	*DAZAP1*	3	*HIST1H3I*	5	*MYLK3*	3	*PTPRT*	4	*TNR*	3
ARID1A	3,4,5	*DCAF8L1*	3	*HIST1H4H*	5	*MYO10*	3	*PTPRU*	3	*TNRC6B*	8

ARID1B	3	*DCBLD1*	5	*HIST2H2BE*	3	*MYO15A*	3	*PTPRZ1*	3	*TNS1*	5
ARID5B	3	*DCDC5*	5	*HK3*	4	*MYO5C*	4	*PXK*	4	*TOX*	3
ARMC4	3	*DCHS1*	1,2	*HLA-A*	3	*MYOM2*	1,2	*RAB11FIP5*	3	*TP53*	1,2,3,4, 5,6,7,8,9
ARSD	3	*DCP1B*	8	*HLA-B*	3	*MYRIP*	4	*RAB3GAP1*	3,4	*TP73*	5
ARSF	3	*DCX*	5	*HMCN1*	3,5	*MYT1L*	3	*RAC2*	3	*TPP2*	3
AS3MT	3	*DDB1*	4	*HNF1B*	1,2	*NAGA*	3	*RANBP17*	3	*TPRKB*	4
ASH1L	3	*DDX10*	4	*HNRNPR*	4	*NAV2*	3	*RANBP3L*	3	*TRAF2*	7
ASPM	3	*DDX3X*	5	*HNRNPUL2*	3	*NAV3*	3	*RANBP6*	3	*TRAF3*	1,2
ASTN2	3	*DDX60*	5	*HSF2*	4	*NDUFS1*	4	*RAPGEF2*	4	*TRANK1*	3
ASXL3	3	*DENND3*	5	*HSPD1*	3	*NEB*	3,5	*RB1*	7,9	*TRIM2*	3
ATIC	4	*DENND5A*	3	*HSPG2*	3	*NEDD4*	3	*RBM15B*	4	*TRIM37*	4
ATM	6,7,8,9	*DGKB*	3	*HUWE1*	3	*NEK10*	3	*RBM24*	5	*TRIP11*	4,5
ATMIN	7	*DGKD*	3	*HYDIN*	3	*NELL1*	5	*RBM38*	3	*TROAP*	3
ATP10A	3,4	*DHDH*	9	*ID3*	5	*NELL2*	4	*RBP3*	3	*TRPC1*	3
ATP10B	3	*DHX15*	3	*IDH1*	4	*NF1*	4	*RC3H2*	3	*TRPC4*	5
ATP11C	3,9	*DHX33*	3	*IGF1R*	3	*NFIA*	3	*RCE1*	3	*TRPM6*	8
ATP1A2	4	*DIAPH2*	3	*IGHMBP2*	3	*NFIB*	4	*REG1B*	3	*TRPS1*	3
ATP2A1	5	*DIAPH3*	4	*IGLL5*	5	*NFKBIA*	3	*RELN*	3,4	*TRRAP*	4
ATP2A3	4	*DIP2B*	4	*IGSF9B*	3	*NFKBIE*	3	*REV1*	3	*TSC2*	3,4
ATP8B4	5	*DISP1*	3	*IKZF3*	3,5	*NFX1*	4	*RFC1*	4	*TSC22D1*	1,2
ATP9B	3	*DLC1*	3,9	*IL31*	3	*NGFR*	4	*RFTN1*	3,5	*TSC22D2*	3
ATRN	3	*DLGAP1*	3,4	*IL4R*	3	*NIN*	9	*RFXANK*	5	*TSHZ2*	4
ATRNL1	3	*DLGAP2*	5,8	*INADL*	3	*NIPBL*	3,4	*RGS1*	4	*TSKS*	3
ATRX	3	*DMBT1*	3	*INO80*	3	*NKRF*	3	*RGS12*	3	*TTC18*	3
ATRX	5	*DMD*	3,5	*INSC*	4	*NLGN1*	3	*RGS4*	8	*TTC7B*	3
AURKC	3	*DMXL1*	3,4	*INSR*	3	*NLGN2*	4	*RHOA*	4	*TTLL8*	3
B2M	1,2,3, 4,5	*DNAH1*	3	*INTS3*	5	*NLRC4*	5	*RIMS1*	5	*TTN*	3,5,8
B4GALNT2	4	*DNAH10*	5	*INTS6*	4	*NLRP7*	4	*RIMS2*	3	*TUSC3*	3
BAI3	3,5	*DNAH11*	3	*IQGAP1*	4	*NLRP8*	3	*RINT1*	3	*TYRO3*	3
BAZ1A	4	*DNAH5*	3,5	*IQUB*	4,5	*NOC3L*	5	*RIPK4*	5	*UBE2A*	3,5
BAZ2B	3	*DNAH6*	5	*IRF8*	3,4	*NOL8*	3	*RNF123*	3	*UBR5*	3,7,8,9
BCAT2	4	*DNAH7*	3,5	*IRS4*	3	*NOTCH1*	1,2,3,6, 7,8,9	*RNF128*	3	*UGGT2*	3
BCL11A	3	*DNAH9*	3,5	*ITGA2*	4,5	*NOTCH2*	3,4,5,8	*RNF17*	5	*UGT1A10*	3
BCL2	1,2,3,4	*DNAJC6*	8	*ITGA8*	3,4	*NOTCH4*	3	*RNF214*	3	*UGT2B7*	5
BCL6	3,4,5	*DOCK2*	4	*ITGA9*	3	*NPAS3*	5	*RNF40*	3	*UHRF1BP1L*	4
BCL7A	4	*DOCK4*	5	*ITGAE*	3	*NPHP1*	4	*ROBO1*	3,5	*UNC13B*	4
BCL9L	3	*DOK5*	3	*ITGAV*	5	*NPY2R*	4	*ROBO2*	3,5,9	*UNC13C*	5
BCR	3	*DOLK*	4	*ITGB3*	4	*NR1H4*	5	*ROR1*	3	*UNC5A*	5
BHMT2	3	*DOPEY1*	3	*ITIH2*	3	*NRAP*	3	*ROR2*	3,4	*UNC5C*	3
BIRC3	8,9	*DPP10*	5	*ITPKB*	3	*NRK*	3	*RPAP2*	3	*UNC5D*	3,4
BIRC6	3	*DPY19L2*	3	*ITPR3*	3	*NRK*	5	*RPAP3*	3	*UNC80*	5,8
BLM	4	*DPYD*	1,2,3	*ITSN2*	3	*NRXN1*	3	*RPGR*	3	*UQCRC1*	4
BMPER	3	*DRD1*	5	*JAG1*	3,4	*NRXN2*	4	*RPGRIP1L*	5	*USH2A*	3,5
BMPR1A	4	*DROSHA*	5	*JAKMIP2*	4	*NRXN3*	3,5	*RPL10*	3	*USP17L2*	3
BNC2	3	*DSC1*	3	*JPH3*	5	*NSD1*	4	*RSL1D1*	4	*USP7*	3
BNIP3	3	*DSC2*	4	*KBTBD6*	3	*NTS*	5	*RTTN*	3	*VAX1*	3
BPTF	3	*DSC3*	1,2	*KCNA3*	4	*NUP160*	3	*RUNDC1*	4	*VCAN*	3

BRAF	3	*DSCAM*	3	*KCNAB3*	4	*NUP205*	3	*RWDD3*	3	*VMO1*	3
BRCA2	3	*DSCAML1*	4	*KCNC2*	3,8	*OBSCN*	3,5	*RYR1*	3,4	*VMP1*	5
BRCA2	4	*DSEL*	3	*KCNH7*	5	*ODZ1*	3	*RYR2*	3,5	*VPS13A*	4
BRD4	4	*DSG2*	4	*KCNJ12*	5	*ODZ2*	3	*S1PR2*	3	*VPS13D*	5
BRWD1	3,5	*DSP*	4	*KCNJ18*	5	*ODZ3*	3	*SALL3*	9	*VSIG8*	3
BRWD3	3	*DST*	3,5	*KCNJ6*	4	*ODZ3*	5	*SAMD4A*	3	*VWF*	3
BSCL2	4	*DTX1*	3,4,5	*KCNQ3*	3	*ODZ4*	3	*SAPS3*	4	*WAC*	3,5
BSN	3,5	*DUOXA2*	4	*KCNT2*	3,4	*OFD1*	1,2	*SART3*	3	*WDFY3*	5
BTAF1	4	*DUPD1*	4	*KCNV1*	3	*OGDHL*	9	*SCN10A*	5	*WDR65*	4
BTBD12	3	*DUSP2*	3	*KCTD16*	3	*OLIG3*	3	*SCN7A*	3	*WDR66*	4
BTBD3	4	*DUSP27*	1,2	*KCTD3*	3	*ONECUT1*	4	*SCN9A*	3	*WDR67*	4
BTG1	3,4,5	*DYNC1H1*	3,4,5	*KDELC2*	3	*OPHN1*	3	*SCYL1*	4	*WDR7*	5
BTG2	5	*DYNC1I1*	4	*KDM2B*	1,2	*OPN4*	3	*SEC14L1*	3	*WDSUB1*	3
BTK	3	*DYNC2H1*	3	*KDM3B*	3	*OR10A2*	5	*SEMA3A*	3,4	*WEE1*	3
C10orf111	3	*DYRK1A*	3	*KIAA0020*	3	*OR10G3*	3	*SEMA3C*	3	*WHSC1*	4,8,9
C10orf81	3	*EBF1*	3,5	*KIAA0430*	3	*OR10H2*	3	*SEMA3D*	4	*WIF1*	4
C10orf90	3	*ECGF1*	4	*KIAA0556*	3	*OR14A16*	5	*SEMA3E*	3	*WNK1*	3
C12orf35	1	*EEF1A1*	5	*KIAA1109*	3	*OR1J2*	3	*SEMA5A*	3,4	*WNK4*	4
C12orf35	2	*EEFSEC*	3	*KIAA1383*	5	*OR2L2*	5	*SERINC2*	4	*WT1*	4
C15orf2	3	*EIF2AK4*	9	*KIAA1432*	3	*OR2W5*	3	*SETD2*	3,4	*WWC3*	3
C1orf175	3	*EIF4A2*	5	*KIAA1549*	3	*OR4E2*	3	*SETD5*	5	*XDH*	4
C20orf117	3	*EIF4ENIF1*	3	*KIAA1614*	3	*OR4K1*	3	*SETDB1*	3	*XIRP1*	3
C20orf132	3	*ENPP7*	5	*KIAA1671*	8	*OR52E2*	5	*SF3B1*	3	*XIRP2*	3
C9	4	*ENTPD4*	5	*KIAA2022*	5	*OR56A1*	3	*SFMBT2*	5	*XKR6*	5
C9orf79	3	*EP300*	1,2,3	*KIF1B*	3	*OR5D13*	3	*SFTPD*	3	*XPO1*	3
CACNA1A	3	*EP400*	5	*KIF21B*	4	*OR5T2*	3	*SGK1*	3,4,5	*YLPM1*	3,5
CACNA1E	3	*EPHA3*	4	*KIF2B*	3	*OR6K3*	3	*SH3PXD2B*	4	*ZBTB7B*	3
CACNA1G	3	*EPHA5*	4	*KIF4B*	3	*OR8H2*	3	*SH3RF2*	3	*ZC3H12A*	3
CACNA2D1	3	*EPHA7*	3,4	*KLB*	4	*OR8J1*	3	*SH3TC2*	3	*ZC3H18*	3
CACNA2D3	3	*EPS8L2*	3	*KLC3*	4	*OSBPL10*	4	*SHANK3*	3	*ZFHX3*	3
CAD	4	*ERBB3*	4	*KLF2*	1,2,5	*OSBPL1A*	3	*SHKBP1*	3	*ZFHX4*	3
CADM2	3	*ERBB4*	3	*KLF4*	4	*OXR1*	3	*SHMT2*	4	*ZFP106*	3
CADPS	3	*ERC1*	5	*KLHDC2*	3	*P2RY8*	3	*SHROOM2*	3	*ZFP28*	5
CADPS	5	*ERCC6*	3	*KLHL14*	3,4	*PABPC1*	3	*SI*	3,9	*ZFP36L1*	3,5
CALD1	3	*ERN2*	4	*KLHL25*	3	*PABPC5*	5	*SIGLEC1*	3	*ZIC4*	4
CAMK2D	5	*ESX1*	9	*KLHL38*	3	*PADI1*	4	*SIGLEC10*	4	*ZMYM1*	5
CAPN5	4	*ESYT3*	3	*KLHL4*	3	*PADI4*	3	*SKA3*	5	*ZMYM4*	3
CAPN6	5	*ETS1*	5,7	*KLHL6*	3	*PAPLN*	5	*SLC16A7*	4	*ZNF117*	9
CAPN7	4	*ETV6*	3	*KRAS*	3	*PAPPA2*	4	*SLC17A4*	3	*ZNF142*	3
CARD11	1,2,3,4	*EVPL*	3	*KRT24*	3	*PARP6*	5	*SLC17A6*	8	*ZNF208*	3,5
CASP7	3	*EXOC6B*	5	*KRT3*	3	*PASD1*	3	*SLC22A16*	3	*ZNF292*	5
CCDC132	4	*EXTL3*	4	*KRT6A*	4	*PBX1*	4	*SLC26A9*	3	*ZNF296*	9
CCDC46	4	*EYA4*	5	*KRTAP5-5*	3	*PCCB*	4	*SLC35F1*	5	*ZNF311*	3
CCDC88A	3	*EZH2*	1,2,3	*LAMA1*	3,5	*PCDH10*	3	*SLC38A8*	3,4	*ZNF335*	3
CCDC97	4	*FAM13B*	3	*LAMA2*	3	*PCDH11X*	3	*SLC45A4*	3	*ZNF354A*	4
CCND1	6,7,8,9	*FAM47B*	3	*LAMA3*	3	*PCDH15*	3,5	*SLC4A7*	3	*ZNF366*	4
CCND3	1,2,3	*FAM55C*	5	*LAMB1*	3	*PCDH17*	3	*SLC4A8*	4	*ZNF410*	3
CCNF	4	*FAM5C*	3	*LAPTM5*	5	*PCDH20*	3	*SLC5A1*	4	*ZNF439*	4
CCT8L2	5	*FAM62C*	4	*LCA5*	3	*PCDH7*	3,4	*SLC5A12*	5	*ZNF462*	3
CD14	7	*FAM83B*	5	*LCT*	3	*PCDHA6*	3	*SLC6A12*	3	*ZNF474*	4

CD163L1	3,5	*FANCD2*	4	*LEPREL1*	4	*PCDHB1*	4	*SLC6A15*	3	*ZNF493*	3
CD28	5	*FAS*	5	*LIFR*	4,5	*PCDHB15*	4	*SLC9A5*	4	*ZNF518A*	5
CD36	1,2,5	*FASN*	3	*LIG3*	3	*PCDHB2*	5,9	*SLCO1A2*	5	*ZNF521*	3
CD58	1,2,3	*FAT1*	3,5	*LILRA2*	4	*PCDHB3*	3,4	*SLCO1C1*	3	*ZNF598*	3
CD70	3	*FAT2*	3	*LILRB5*	3	*PCDHB5*	4	*SLITRK1*	3	*ZNF600*	3
CD79B	1,2,3,<br>4,5	*FAT3*	3	*LIN7C*	4	*PCDHB6*	3	*SLITRK3*	5	*ZNF608*	3,5
CD83	3	*FAT3*	5	*LIPE*	3	*PCDHGA2*	4	*SLITRK5*	3	*ZNF662*	5
CD93	3	*FAT4*	3,5,9	*LOC153328*	3	*PCDHGB4*	3	*SLITRK6*	3,5	*ZNF676*	3
CDC123	4	*FBN2*	3	*LPHN2*	3,4,5	*PCLO*	3,5	*SMARCA4*	9	*ZNF700*	4
CDC42BPB	3	*FBXO11*	3	*LPHN3*	5	*PCSK2*	8	*SMC1A*	9	*ZNF729*	5
CDH10	4,5	*FBXW2*	3	*LPIN3*	3	*PCSK4*	3	*SMEK1*	4	*ZNF77*	3
CDH11	3	*FBXW7*	3,4	*LRAP*	4	*PCSK5*	5	*SMG6*	3	*ZNF770*	3
CDH12	3	*FCRL5*	3	*LRBA*	3,5	*PDE10A*	3	*SNRNP40*	3	*ZNF790*	4
CDH18	3	*FGD3*	4	*LRFN2*	3	*PDE1C*	4	*SOCS1*	3,4	*ZNF804A*	3,5
CDH19	3	*FGF10*	3	*LRIG3*	3,4,5	*PDE3A*	3	*SOCS5*	5	*ZNF804B*	3
CDH22	3	*FGFR1*	4	*LRP1*	4,5	*PDE4B*	5	*SON*	5	*ZNF830*	3
CDH7	4	*FGFR2*	3	*LRP10*	3,4	*PDE4DIP*	5	*SORCS2*	3	*ZNF91*	4
CDH8	4,9	*FHIT*	3	*LRP1B*	3,4,5,8	*PDE8B*	5	*SOX6*	4	*ZP1*	4
CDK3	4	*FIGN*	4,5	*LRP2*	5	*PDGFC*	3	*SP140*	8	*ZP3*	3
CDKN2A	5,7	*FLG*	3,5	*LRP3*	4	*PDGFRA*	4	*SP3*	3		
CDR1	5	*FLNC*	3,4,8	*LRRC49*	5	*PDIA2*	4	*SPAST*	3		

Ref., reference; Reference 1 = Pasqualucci et al., 2011 [4]; Reference 2 = Morin et al., 2011 [5]; Reference 3 = Lohr et al., 2012 [6]; Reference 4 = Zhang et al., 2013 [7]; Reference 5 = de Miranda et al., 2014 [8]; Reference 6 = Kridel et al., 2012 [9]; Reference 7 = Meissner et al., 2013 [10]; Reference 8 = Bea et al., 2013 [11]; Reference 9 = Zhang et al., 2014 [12].

Supplementary Table S5: Recurrently mutated genes in MCL tumors in the present study

ID	*Gene*	Somatic or not	Patients ID	SNVs	SIFT	Polyphen2	In COSMIC	Function
1	*ATM*	Somatic<br>Somatic<br>Somatic<br>Somatic<br>Somatic<br>Somatic	P4-P,R<br>P5-P,R<br>P7-P<br>P7-P,R<br>P10-<br>R1,R2,R3<br>P12-P	c.G9023A:p.R3008H<br>c.G9023A:p.R3008H<br>c.G6188A:p.G2063E<br>c.7760_7761insG:p.<br>V2587fs<br>c.C7466G:p.S2489C<br>c.A6056G:p.Y2019C	Damaging<br>Damaging<br>Damaging<br>N.A.<br>Damaging<br>Damaging	Pro.D<br>Pro.D<br>Damaging<br>N.A.<br>Pro.D<br>Pro.D	Yes<br>Yes<br>Yes<br><br><br>Yes	DNA damage response; cell cycle; G2 DNA damage checkpoint [13]
2	*S1PR1*	Somatic<br>Somatic	P6-P<br>P9-<br>P,R1,R2	c.T209G:p.I70S<br>c.363_364insG:<br>p.E121fs	Damaging<br>N.A.	Pro.D<br>N.A.		Crucial for persistent STAT3 activation in tumor cell migration [14, 15]
3	*MEF2B*	Somatic<br>Somatic<br>Somatic	P6-P,R<br>P9-<br>P,R1,R2<br>P12-P	c.A146G:p.N49S<br>c.A68G:p.K23R<br>c.A68G:p.K23R	Damaging<br>Damaging<br>Damaging	Pro.D<br>Pro.D<br>Pro.D		Chromatin modification; transcriptional activator; mutated in DLBCL, MCL, FL [11, 16]

4	*ARHGAP32*	Somatic<br>Somatic	P5-P,R<br>P7-P,R	c.A869G:p.D290G<br>c.C2051T:p.P684L	Damaging<br>Damaging	Pos.D<br>Pro.D		N-cadherin/beta-catenin complex in the ER and ER exit site; downregulation resulting in a decrease in cell-cell adhesion [17]
5	*SLITRK5*	Somatic<br>Somatic	P4-R<br>P9-P,R1,R2	c.C1703A:p.T568N<br>c.G1204A:p.A402T	Damaging<br>Damaging	Benign<br>Benign		Neuron-specific transmembrane protein; homology with neurotrophin receptors in C-terminal regions [18]
6	*CARD11*	Somatic<br>Somatic	P5-P,R<br>P9-R2	c.A644T:p.K215M<br>c.G688A:p.D230N	Damaging<br>Tolerated	Pro.D<br>Pro.D	 Yes	BCR signaling; activates NF-κB via BCL10 and IKK [19]
7	*PPIG*	Somatic<br>Somatic	P4-P,R<br>P6-P	c.T206A:p.V69D<br>c.G743A:p.R248Q	Damaging<br>Tolerated	Pro.D<br>Pos.D		May be involved in folding, transport, and assembly of proteins; regulation of pre-mRNA splicing
8	*ACTN2*	Somatic<br>Somatic	P6-P<br>P12-P	c.C2147G:p.T716R<br>c.T437C:p.I146T	Damaging<br>Damaging	Pro.D<br>Pro.D		F-actin cross-linking protein, involved in binding actin to the membrane [20]
9	*PLXNA1*	Somatic<br>Somatic	P9-R1<br>P13-P	c.G4795T:p.A1599S<br>c.G391A:p.G131S	Tolerated<br>Damaging	Pro.D<br>Pro.D		Regulation of the immune system, involved in the survival of cell line [21]
10	*CCM2L*	P.S.<br>Somatic	P2-R<br>P5-P	c.G1108A:p.A370T<br>c.G1298A:p.C433Y	Damaging<br>Damaging	Benign<br>Pro.D		Cardiovascular development, necessary component of the Heart of Glass-Cerebral Cavernous Malformation (Heg-CCM) pathway [11]
11	*CHSY3*	P.S.<br>Somatic	P3-R2<br>P4-P,R	c.C1675T:p.R559C<br>c.G1253A:p.R418H	Damaging<br>Tolerated	Pro.D<br>Benign	Yes	Glycosyl transferase; chondroitin polymerization
12	*PPM1D*	P.S.<br>Somatic	P3-R2<br>P6-R	c.G428A:p.R143H<br>c.1344delT:p.N448fs	Damaging<br>N.A.	Damaging<br>N.A.		Oncogene, promotes termination of the DDR pathway by inactivating p53 [22]

13#	*PKHD1*	P.S.<br>Somatic	P8-R2<br>P10-R1,R2,R2	c.G2639A:p.R880H<br>c.G8672A:p.R2891H	Damaging<br>Damaging	Pro.D<br>Pos.D		Mutation causes autosomal recessive polycystic kidney disease [23]
14#	*TTN*	P.S.<br><br>Somatic	P3-R2<br><br>P10-R1,R2,R3	c.67328_67343del:p.22443_22448del<br>c.G4025T:p.G1342V	N.A.<br><br>Tolerated	N.A.<br><br>Benign		Key component in the assembly and functioning of vertebrate striated muscles [24]
15#	*FAT1*	P.S.<br>P.S.<br>P.S.	P1-P,R<br>P3-R1,R2<br>P8-P,R1,R2	c.G11003T:p.R3668L<br>c.C1031T:p.P344L<br>c.G2734A:p.V912I	Damaging<br>Damaging<br>Tolerated	N.A.<br>N.A.<br>N.A.	 <br>Yes	Tumor suppressor gene, and recurrent mutation in multiple human cancers<br>leads to aberrant Wnt activation [25]
16	*DGKB*	Somatic	P5-P,R<br>P8-P,R1,R2	c.1170+2T>C<br>c.C488T:p.T163M	N.A.<br>Damaging	N.A.<br>N.A.		Diacylglycerol kinase, play a key role in cellular processes
17#	*PCLO*	P.S.<br>Somatic	P3-R1,R2<br>P9-P,R1,R2	c.C10849T:p.P3617S<br>c.C10943A:p.P3648H	Damaging<br>Damaging	N.A.<br>N.A.		Somatic mutated in DLBCL; may act as a scaffolding protein involved in the organization of synaptic active zones and in synaptic vesicle trafficking [6]
18	*YLPM1*	P.S.<br>Somatic	P2-P,R<br>P9-R1	c.C1891T:p.P631S<br>c.338_339insG:p.P113fs	Damaging<br>N.A.	N.A.<br>N.A.		ZAP3, a putative nucleoside kinase nuclear, interacts with protein phosphatase (PP1) [26]
19#	*DNAH5*	P.S.<br>P.S.	P8-R2<br>P11-P,R	c.G6748A:p.E2250K<br>c.G6748A:p.E2250K	Damaging<br>Damaging	Damaging<br>Damaging	Yes<br>Yes	Significantly mutated genes in Colon cancer [27]; functions as a force-generating protein with ATPase activity
20	*MLL2*	P.S.<br>P.S.<br>P.S.	P1-P,R<br>P8-P,R1,R2<br>P11-P,R	c.C12664G:p.L4222V<br>c.C4895A:p.S1632X<br>c.969_970insGTGTG:p.C323fs	N.A<br>N.A.<br>N.A..	N.A.<br>N.A.<br>N.A.		Chromatin modifier, mutated in MCL, FL, DLBCL [6, 11, 28]
21#	*FLG*	P.S.<br>P.S.	P2-P,R<br>P3-R1,R2	c.A9773T:p.H3258L<br>c.T683C:p.I228T<br>c.G3850T:p.E1284X	Damaging<br>Tolerated<br>N.A.	Benign<br>Benign<br>N.A.		Loss-of-function mutations in the FLG cause ichthyosis vulgaris [29]

22#	*ABCA13*	P.S.<br>P.S.	P3-R1,R2<br>P11-P,<br>P11-R	c.T9361G:p.C3121G<br>c.T2309C:p.I770T	Damaging<br>Tolerated	N.A.<br>N.A.		ATP-binding cassette (ABC) family member, and its mutation was frequent found in Renal Cell Cancer [30]
23	*ANKRD17*	P.S.<br>P.S.	P3-R1,R2<br>P11-P,R	c.A7045C:p.N2349H<br>c.303_314del:p.101_105del	Damaging<br>N.A.	Benign<br>N.A.		Positive regulator of the RIG-I-like receptor (RLR)-mediated immune signaling [31]
24	*RIMS2*	P.S.<br>P.S.	P2-P,R<br>P3-R1,R2	c.G1265T:p.G422V<br>c.C2081T:p.S694L	Damaging<br>Damaging	Damaging<br>Damaging		Functions in exocytosis, and is essential for neurotransmitter release [32]
25	*GON4L*	Somatic<br>P.S.	P7-P<br>P1-P,R	c.C92T:p.A31V<br>c.A1315G:p.I439V	Damaging<br>Tolerated	Benign<br>Benign		Regulates gene expression during hematopoietic development [33]; essential for B lymphopoiesis [34]

Genes with ID 1–9 are carrying somatic mutations from cases with control counterparts; Genes with ID 10–25 (shaded in dark gray) are additional genes affected when including the "somatic variants" identified from tumor paired-comparison in cases without controls. P.S, putatively somatic; Pos.D: possible damaging; Pro.D: probably damaging; P1, patient No.1; P, primary tumor; R, relapse tumor; R1, first relapse tumor; R2, second relapse tumor; R3, third relapse tumor; N.A., not applicable; #, Genes that are highly mutated in human germline genome were not included in Figure 1C.

Supplementary Table S6: Summary of variations discovered in *CARD11* gene in a Swedish MCL cohort (*n* = 200 samples from 179 patients, including samples characterized by WES)

Gene	Nucleotide change	AA change	Found in Exome	Found in Sanger	Mutated tumor	Novel or not	SIFT [35]	Polyphen2 [36]
CARD11	c.367 G > A	p.G123S		Yes	1 (P247)	Novel	Damaging	Damaging
	c.377 G > A	p.G126D		Yes	1 (P239)	Novel	Damaging	Damaging
	c.644 A > T	p.K215M	Yes	Yes	2 (P5P, P5R)	Novel	Pro. Damaging	Damaging
	c.645 G > C	p.K215N		Yes	1 (P231)	Novel	Damaging	Damaging
	c.688 G > A	p.D230N	Yes	Yes	1 (P9)	Novel	Pro. Damaging	Damaging
	c.688 G > A	p.D230N		Yes	1 (P73)	Novel	Pro. Damaging	Damaging
	c.1071 C > A	p.D357E		Yes	1 (P71)	Novel	Pro. Damaging	Benign
	c.1078 A > G	p.M360V		Yes	1 (P122)	Novel	Tolerated	Benign
	c.1082 A > G	p.Y361C		Yes	1 (P20)	Novel	Pro. Damaging	Damaging
	c.1082 A > G	p.Y361C		Yes	1 (P97)	Novel	Pro. Damaging	Damaging

Pro.Damaging, Probably Damaging; AA, amino acid.

Supplementary Table S7: Characteristics of patients with *CARD11* genetic alterations

	Total	*CARD11* Wild-type	*CARD11* mutated	*p* value (*CARD11*)
**Number of patients**	179	169	10	
**Age at diagnosis**				
Median, year	69.3	69.2	72.5	
**Gender**				
Male	132	74.0% (125/169)	70.0% (7/10)	1.0000
Female	47	26.0% (44/169)	30.0% (3/10)	1.0000
**Features and outcome**				
Stage IV at diagnosis[A]	81.7% (134/164)	81.4% (127/156)	87.5% (7/8)	1.0000
Blastoid variants[B]	18.9% (37/196)	18.9% (35/185)	18.2% (2/11)	0.9636
Relapse samples[C]	23.6% (47/199)	22.9% (43/188)	36.4% (4/11)	0.2190
Overall survival, months[D]	43.3	43.2	45.1	0.6091
Status, dead	63.1% (111/176)	63.1% (106/168)	60% (6/10)	N.A.

The table combines the clinical data from the discovery cohort (27 samples from 13 patients that underwent WES) and the screening cohort (179 samples from 170 patients that characterized by Sanger sequencing). Note that 6 samples (from 4 patients) out of those 27 samples underwent WES were also screened by Sanger sequencing. Altogether, 200 MCL samples from 179 Swedish patients were included. A-D, the calculation was based on 164, 196, 199 or 176 samples with available data; N.A., not applicable. The statistical analysis was performed by χ^2 test. *$p < 0.05$.

Supplementary Table S8: Mutations identified in genes belonging to BCR or NF-κB pathway in MCL cell lines

Cell line	Gene	SNVs	SIFT	Polyphen2	Cosmic	Function and Pathway
Granta519	*IKBKG*#	c.G343A:p.A115T	Tolerated	Benign	Yes	BCR-NF-κB
JVM2	*TRAF1*#	c.T50C:p.M17T	Tolerated	Benign	Yes	NIK-NF-κB
	MAP3K7	c.T1175A:p.M392K	Damaging	Damaging	Yes	NF-κB
Rec-1	*NFKBIB*#	c.G212A:p.R71H	Damaging	Damaging	Yes	BCR-NF-κB; inhibits NF-κB by complexing with and trapping it in the cytoplasm
Mino	*NFKBIB*	c.C199T:p.R67C	Tolerated	Benign	Yes	BCR- NF-κB
	PLAU#	c.G43T:p.V15L	Damaging	Possible damaging	Yes	BCR-NF-κB; cell migration and invasion
	TRIM25#	c.C1469T:p.P490L	Possible damaging	Benign	Yes	NF-κB; ubiquitin E3 ligase and as an ISG15 E3 ligase
	BCL6#	c.G492T:p.E164D	Tolerated	Benign	Yes	BCR; augments BCR signaling
	NRAS#	c.G38A:p.G13D	Damaging	Damaging	Yes	BCR; Ras proteins bind GDP/GTP and possess intrinsic GTPase activity; oncogene
Jeko-1	*PIP5K1C*	c.G1010A:p.G337D	Tolerated	Benign	Yes	BCR; endocytosis and cell migration; negative regulator of T-cell activation and adhesion
	PLAU	c.550_551insG:p.R184fs	N.A.	N.A.	Yes	BCR-NF-κB
	CR2#	c.G641A:p.R214H	Possible damaging	Benign	Yes	BCR; receptor for complement C3Dd, for the Epstein-Barr virus on human B-cells and T-cells;participates in B lymphocytes activation
Z-138	*TRAF2*	c.G341A:p.W114X<br>c.G125A:p.R42H	N.A.<br>Damaging	N.A.<br>Damaging	Yes	NIK-NF-κB
	PIDD#	c.C1954T:p.R652W	Damaging	Damaging	Yes	NF-κB; promotes apoptosis; regulated by p53; enhances sumoylation and ubiquitination of NEMO/IKBKG
	MAP3K14#	c.G419A:p.S140N	Tolerated	Damaging	Yes	NIK-NF-κB; as kinase binds to TRAF2 and stimulates NF-κB activity
	NFKBIZ#	c.A1168G:p.M390V	Tolerated	Benign	Yes	BCR-NF-κB
	ICAM1	c.C1519T:p.R507W	Damaging	Damaging	Yes	NIK-NF-κB; cell surface glycoprotein

N.A., not applicable; NIK (MAP3K14), a key component in alternative NF-κB pathway; Genes labeled with # indicate the variants found in these genes are rare SNPs (< 1% in allele frequency).

Supplementary Table S9: Signaling pathways targeted in relapsed MCL

Gene	Sample	SNVs	SIFT	Polyphen2	Webgestalt Pathway	Function or pathway#
PAK1	P9-R1	c.C1003T:p.L335F	Damaging	Pro.Damaging	TCR; Wnt; MAPK	T cell receptor signaling
PLCG1	P7-R	c.C1973T:p.P658L	Damaging	Pro.Damaging	TCR	T cell receptor signaling; leukocyte migration; ERBB signaling
CARD11	P9-R2	c.G688A:p.D230N	Tolerated	Pro.Damaging	TCR; BCR	B- and T- cell receptor signaling; NF-κB
CD28	P7-R	c.G222C:p.Q74H	Tolerated	Benign	TCR	T cell receptor signaling, survival of plasma cells
AKT2	P6-R	c.C322T:p.R108W	Damaging	Pro.Damaging	TCR; MAPK	T cell receptor signaling, promote B cell survival
MAPK3	P4-R	c.G484T:p.V162L	Damaging	Pos.Damaging	TCR; MAPK	T cell receptor signaling
PPM1D	P3-R2<br>P6-R	c.G428A:p.R143H<br>c.1344delT:p.N448fs	Damaging<br>N.A.	Pro.Damaging<br>N.A.	p53	Oncogene; phosphorylate and stabilize MDM2 (negative feedback of p53-MDM2); relief of p53-dependent checkpoint mediated cell cycle arrest; phosphorylate and inactivate CHEK1; suppresses apoptosis [22]
GTSE1	P4-R	c.C554T:p.A185V	Tolerated	Benign	p53	p53 pathway; antiapoptotic; cell migration
CCNB3	P6-R	c.G331A:p.A111T	Damaging	Benign	P53	p53 pathway; cyclinB3, positive regulatory subunits of the cyclin-dependent kinases
TP53	P8-R2	c.435_439del: p.145_147del	N.A.	N.A.	p53; MAPK	DNA repair; cell cycle; tumor suppressor
CCDC8	P9-R1	c.G390T:p.M130I	Damaging	Pro.Damaging	p53#	Cofactor required for p53-mediated apoptosis when DNA damage; play a role in growth through interactions with the cytoskeletal adaptor protein obscurin-like 1 [37]
GLI3	P7-R	c.C370G:p.P124A	Tolerated	Benign	Wnt#, p53#	Activate MDM2-p53 pathway; suppress growth of colon cancer cells; a repressor of the sonic hedgehog pathway; transcriptional activator [38, 39]
NTRK2	P7-R	c.G1241A:p.S414N	Damaging	Benign	MAPK	MAPK signaling
DUSP5	P11-R	c.C879G:p.I293M	Damaging	Benign	MAPK	MAPK signaling
MAPK8IP3	P3-R2	c.C349G:p.Q117E	Tolerated	Pro.Damaging	MAPK	MAPK signaling

Signaling pathways that are significantly ($p < 0.01$) targeted in relapse MCLs are shown in this table. Pathway analysis was performed on Webgestalt (WEB-based GEne SeT AnaLysis Toolkit). Pos.Damaging, possible damaging; Pro.Damaging, probably damaging; P, patient; R, relapse tumor; R1, first relapse tumor; R2, second relapse tumor; N.A., not applicable; TCR, T cell receptor signaling. #, Functional pathway supported in literatures.

Supplementary Table S10: The dominant V(D)J rearrangemnts identified in MCL samples

Sample ID	Tumor cell, %	V	D	J	Identity, %	SHM status
P1-P	90	V3-48*01	D6-6*01	J4*02	86.36	Hypermutated
P1-R	46	V3-48*01	D6-6*01	J4*02	96.69	Significantly mutated
P2-P	50	V3-21*01	D1-1*01	J6*03	98.76	Minimally mutated
P2-R	45	V3-21*01	D1-1*01	J6*03	98.76	Minimally mutated
P3-R1	80	V3-9*01	D6-25*01	J4*02	100	Unmutated
P3-R2	80	V3-9*01	D6-25*01	J4*02	100	Unmutated
P4-P§	80	V1-2*04	D3-22*01	J4*02	99.54	Minimally mutated
P4-R§	90	V1-2*04	D3-22*01	J4*02	99.54	Minimally mutated
P5-P	80	V3-23*04	D2-15*01	J4*02	95.87	Significantly mutated
P5-R	80	V3-23*04	D2-15*01	J4*02	95.87	Significantly mutated
P6-P	86	V4-34*01	D6-25*01	J5*02	100 or 99.54	Unmutated (86%); Minimally mutated (14%)
P6-R	86	V4-34*01	D6-25*01	J5*02	100 or 99.54	Unmutated (75%); Minimally mutated (25%)
P7-P	86	V3-53*01	D6-19*01	J4*02	97.91	Minimally mutated
P7-R	70	V3-53*01	D6-19*01	J4*02	97.91	Minimally mutated
P8-P	66	V3-23*04	D2-21*02	J4*02	100	Unmutated
P8-R1	81	V3-23*04	D2-21*02	J4*02	100	Unmutated
P8-R2	56	V3-23*04	D2-21*02	J4*02	100	Unmutated
P9-P	80	V4-34*01	D3-10*01	J5*02	100	Unmutated
P9-R1	80	V4-34*01	D3-10*01	J5*02	100	Unmutated
P9-R2	86	V4-34*01	D3-10*01	J5*02	100	Unmutated
P10-R1	63	V4-61*01	D3-3*01	J6*02	100	Unmutated
P10-R2	61	V4-61*01	D3-3*01	J6*02	100	Unmutated
P10-R3	82	V4-61*01	D3-3*01	J6*02	100	Unmutated
P11-P	78	V3-48*01	D2-15*01	J4*02	100	Unmutated
P11-R	80	V3-48*01	D2-15*01	J4*02	100	Unmutated

SHM, somatic hypermutation; unmutated, 100% identical to the respective germline sequence [40]; minimally/borderline mutated, 99.9–97% identical to the corresponding germline sequence; significantly mutated, 96.9–95% identical to the corresponding germline sequence; hypermutated, < 95% identical to the corresponding germline sequence); §, only non-productive IGH rearranged sequences could be found in this tumor.

REFERENCES

1. Yang Y, Shaffer AL 3rd, Emre NC, Ceribelli M, Zhang M, Wright G, Xiao W, Powell J, Platig J, Kohlhammer H, Young RM, Zhao H, Yang Y, et al. Exploiting synthetic lethality for the therapy of ABC diffuse large B cell lymphoma. Cancer cell. 2012; 21:723–737.
2. Kanehisa M, Goto S. KEGG: Kyoto Encyclopedia of Genes and Genomes. Nucleic acids Res. 2000; 28:27–30.
3. Wang J, Duncan D, Shi Z, Zhang B. WEB-based GEne SeT AnaLysis Toolkit (WebGestalt): update 2013. Nucleic acids Res. 2013; 41:W77–83.
4. Pasqualucci L, Dominguez-Sola D, Chiarenza A, Fabbri G, Grunn A, Trifonov V, Kasper LH, Lerach S, Tang H, Ma J, Rossi D, Chadburn A, Murty VV, et al. Inactivating mutations of acetyltransferase genes in B-cell lymphoma. Nature. 2011; 471:189–195.
5. Morin RD, Mendez-Lago M, Mungall AJ, Goya R, Mungall KL, Corbett RD, Johnson NA, Severson TM, Chiu R, Field M, Jackman S, Krzywinski M, Scott DW, et al. Frequent mutation of histone-modifying genes in non-Hodgkin lymphoma. Nature. 2011; 476:298–303.
6. Lohr JG, Stojanov P, Lawrence MS, Auclair D, Chapuy B, Sougnez C, Cruz-Gordillo P, Knoechel B, Asmann YW, Slager SL, Novak AJ, Dogan A, Ansell SM, et al. Discovery and prioritization of somatic mutations in diffuse large B-cell lymphoma (DLBCL) by whole-exome sequencing. Proc Natl Acad Sci U S A. 2012; 109:3879–3884.
7. Zhang J, Grubor V, Love CL, Banerjee A, Richards KL, Mieczkowski PA, Dunphy C, Choi W, Au WY, Srivastava G, Lugar PL, Rizzieri DA, Lagoo AS, et al. Genetic heterogeneity of diffuse large B-cell lymphoma. Proc Natl Acad Sci U S A. 2013; 110:1398–1403.
8. de Miranda NF, Georgiou K, Chen L, Wu C, Gao Z, Zaravinos A, Lisboa S, Enblad G, Teixeira MR, Zeng Y, Peng R, Pan-Hammarstrom Q. Exome sequencing reveals novel mutation targets in diffuse large B-cell lymphomas derived from Chinese patients. Blood. 2014; 124:2544–2553.
9. Kridel R, Meissner B, Rogic S, Boyle M, Telenius A, Woolcock B, Gunawardana J, Jenkins C, Cochrane C, Ben-Neriah S, Tan K, Morin RD, Opat S, et al. Whole transcriptome sequencing reveals recurrent NOTCH1 mutations in mantle cell lymphoma. Blood. 2012; 119:1963–1971.
10. Meissner B, Kridel R, Lim RS, Rogic S, Tse K, Scott DW, Moore R, Mungall AJ, Marra MA, Connors JM, Steidl C, Gascoyne RD. The E3 ubiquitin ligase UBR5 is recurrently mutated in mantle cell lymphoma. Blood. 2013; 121:3161–3164.
11. Bea S, Valdes-Mas R, Navarro A, Salaverria I, Martin-Garcia D, Jares P, Gine E, Pinyol M, Royo C, Nadeu F, Conde L, Juan M, Clot G, et al. Landscape of somatic mutations and clonal evolution in mantle cell lymphoma. Proc Natl Acad Sci U S A. 2013; 110: 18250–8255.
12. Zhang J, Jima D, Moffitt AB, Liu QQ, Czader M, Hsi ED, Fedoriw Y, Dunphy CH, Richards KL, Gill JI, Sun Z, Love C, Scotland P, et al. The genomic landscape of mantle cell lymphoma is related to the epigenetically determined chromatin state of normal B cells. Blood. 2014; 123:2988–2996.
13. Abraham RT. Cell cycle checkpoint signaling through the ATM, ATR kinases. Gene Dev. 2001; 15:2177–2196.
14. Lee H, Deng JH, Kujawski M, Yang CM, Liu Y, Herrmann A, Kortylewski M, Horne D, Somlo G, Forman S, Jove R, Yu H. STAT3-induced S1PR1 expression is crucial for persistent STAT3 activation in tumors. Nat Med. 2010; 16:1421–U1105.
15. Pham TH, Okada T, Matloubian M, Lo CG, Cyster JG. S1P1 receptor signaling overrides retention mediated by G alpha i-coupled receptors to promote T cell egress. Immunity. 2008; 28:122–133.
16. Ying CY, Dominguez-Sola D, Fabi M, Lorenz IC, Hussein S, Bansal M, Califano A, Pasqualucci L, Basso K, Dalla-Favera R. MEF2B mutations lead to deregulated expression of the oncogene BCL6 in diffuse large B cell lymphoma. Nat Immunol. 2013; 14:1084–1092.
17. Nakamura T, Hayashi T, Nasu-Nishimura Y, Sakaue F, Morishita Y, Okabe T, Ohwada S, Matsuura K, Akiyama T. PX-RICS mediates ER-to-Golgi transport of the N-cadherin/beta-catenin complex. Gene Dev. 2008; 22:1244–1256.
18. Shmelkov SV, Hormigo A, Jing D, Proenca CC, Bath KG, Milde T, Shmelkov E, Kushner JS, Baljevic M, Dincheva I, Murphy AJ, Valenzuela DM, Gale NW, et al. Slitrk5 deficiency impairs corticostriatal circuitry and leads to obsessive-compulsive-like behaviors in mice. Nat Med. 2010; 16:598–602.
19. Niemann CU, Wiestner A. B-cell receptor signaling as a driver of lymphoma development and evolution. Semin Cancer Biol. 2013; 23:410–421.
20. Agarwal SK, Simonds WF, Marx SJ. The parafibromin tumor suppressor protein interacts with actin-binding proteins actinin-2 and actinin-3. Mol Cancer. 2008; 7:65.
21. Chen EY, Dobrinski KP, Brown KH, Clagg R, Edelman E, Ignatius MS, Chen JYH, Brockmann J, Nielsen GP, Ramaswamy S, Keller C, Lee C, Langenau DM. Cross-Species Array Comparative Genomic Hybridization Identifies Novel Oncogenic Events in Zebrafish and Human Embryonal Rhabdomyosarcoma. Plos Genet. 2013; 9:e1003727.
22. Kleiblova P, Shaltiel IA, Benada J, Sevcik J, Pechackova S, Pohlreich P, Voest EE, Dundr P, Bartek J, Kleibl Z, Medema RH, Macurek L. Gain-of-function mutations of PPM1D/Wip1 impair the p53-dependent G1 checkpoint. J Cell Biol. 2013; 201:511–521.
23. Fedeles SV, Tian X, Gallagher AR, Mitobe M, Nishio S, Lee SH, Cai YQ, Geng L, Crews CM, Somlo S. A genetic interaction network of five genes for human polycystic kidney and liver diseases defines polycystin-1 as the central determinant of cyst formation. Nat Genet. 2011; 43:639–647.
24. Greenman C, Stephens P, Smith R, Dalgliesh GL, Hunter C, Bignell G, Davies H, Teague J, Butler A, Edkins S, O'Meara S, Vastrik I, Schmidt EE, et al. Patterns of somatic mutation in human cancer genomes. Nature. 2007; 446:153–158.
25. Morris LG, Kaufman AM, Gong Y, Ramaswami D, Walsh LA, Turcan S, Eng S, Kannan K, Zou Y, Peng L, Banuchi VE, Paty P, Zeng Z, et al. Recurrent somatic mutation of FAT1 in multiple human cancers leads to aberrant Wnt activation. Nat Genet. 2013; 45:253–261.
26. Ulke-Lemee A, Laura TMYB, Chaulk S, Bernstein NK, Morrice N, Glover M, Lamond AI, Moorhead GBG. The

nuclear PP1 interacting protein ZAP3 (ZAP) is a putative nucleoside kinase that complexes with SAM68, CIA, NF110/45, and HNRNP-G. Bba-Proteins Proteom. 2007; 1774:1339–1350.

27. Seshagiri S, Stawiski EW, Durinck S, Modrusan Z, Storm EE, Conboy CB, Chaudhuri S, Guan Y, Janakiraman V, Jaiswal BS, Guillory J, Ha C, Dijkgraaf GJ, et al. Recurrent R-spondin fusions in colon cancer. Nature. 2012; 488:660–664.

28. Okosun J, Bodor C, Wang J, Araf S, Yang CY, Pan C, Boller S, Cittaro D, Bozek M, Iqbal S, Matthews J, Wrench D, Marzec J, et al. Integrated genomic analysis identifies recurrent mutations and evolution patterns driving the initiation and progression of follicular lymphoma. Nat Genet. 2014; 46:176–181.

29. Smith FJ, Irvine AD, Terron-Kwiatkowski A, Sandilands A, Campbell LE, Zhao Y, Liao H, Evans AT, Goudie DR, Lewis-Jones S, Arseculeratne G, Munro CS, Sergeant A, et al. Loss-of-function mutations in the gene encoding filaggrin cause ichthyosis vulgaris. Nat Genet. 2006; 38:337–342.

30. Arai E, Sakamoto H, Ichikawa H, Totsuka H, Chiku S, Gotoh M, Mori T, Nakatani T, Ohnami S, Nakagawa T, Fujimoto H, Wang L, Aburatani H, et al. Multilayer-omics analysis of renal cell carcinoma, including the whole exome, methylome and transcriptome. Int J Cancer. 2014; 135:1330–1342.

31. Wang Y, Tong X, Li G, Li J, Deng M, Ye X. Ankrd17 positively regulates RIG-I-like receptor (RLR)-mediated immune signaling. Eur J Immunol. 2012; 42:1304–1315.

32. Kaeser PS, Deng L, Fan M, Sudhof TC. RIM genes differentially contribute to organizing presynaptic release sites. Proc Natl Acad Sci U S A. 2012; 109:11830–11835.

33. Lu P, Hankel IL, Hostager BS, Swartzendruber JA, Friedman AD, Brenton JL, Rothman PB, Colgan JD. The Developmental Regulator Protein Gon4l Associates with Protein YY1, Co-repressor Sin3a, and Histone Deacetylase 1 and Mediates Transcriptional Repression. J Biol Chem. 2011; 286:18311–18319.

34. Lu P, Hankel IL, Knisz J, Marquardt A, Chiang MY, Grosse J, Constien R, Meyer T, Schroeder A, Zeitlmann L, Al-Alem U, Friedman AD, Elliott EI, et al. The Justy mutation identifies Gon4-like as a gene that is essential for B lymphopoiesis. J Exp Med. 2010; 207:1359–1367.

35. Ng PC, Henikoff S. SIFT: predicting amino acid changes that affect protein function. Nucleic Acids Res. 2003; 31:3812–3814.

36. Adzhubei IA, Schmidt S, Peshkin L, Ramensky VE, Gerasimova A, Bork P, Kondrashov AS, Sunyaev SR. A method and server for predicting damaging missense mutations. Nat Methods. 2010; 7:248–249.

37. Dai C, Tang Y, Jung SY, Qin J, Aaronson SA, Gu W. Differential effects on p53-mediated cell cycle arrest vs. apoptosis by p90. Proc Natl Acad Sci U S A. 2011; 108:18937–18942.

38. Villavicencio EH, Walterhouse DO, Iannaccone PM. The sonic hedgehog-patched-gli pathway in human development and disease. Am J Hum Genet. 2000; 67:1047–1054.

39. Kang HN, Oh SC, Kim JS, Yoo YA. Abrogation of Gli3 expression suppresses the growth of colon cancer cells via activation of p53. Experimental cell research. 2012; 318:539–549.

40. Navarro A, Clot G, Royo C, Jares P, Hadzidimitriou A, Agathangelidis A, Bikos V, Darzentas N, Papadaki T, Salaverria I, Pinyol M, Puig X, Palomero J, et al. Molecular Subsets of Mantle Cell Lymphoma Defined by the IGHV Mutational Status and SOX11 Expression Have Distinct Biologic and Clinical Features. Cancer Res. 2012; 72:5307–5316.
